# Consultation rates in people with type 2 diabetes with and without vascular complications: a retrospective analysis of 141,328 adults in England

**DOI:** 10.1186/s12933-021-01435-y

**Published:** 2022-01-10

**Authors:** Sophia Abner, Clare L. Gillies, Sharmin Shabnam, Francesco Zaccardi, Samuel Seidu, Melanie J. Davies, Tobi Adeyemi, Kamlesh Khunti, David R. Webb

**Affiliations:** 1 Leicester Real World Evidence Unit, Diabetes Research Centre, Leicester General Hospital, University of Leicester, Leicester, LE5 4PW UK; 2 Leicester Diabetes Centre, Leicester General Hospital, Leicester, LE5 4PW UK; 3 Leicester Diabetes Centre, National Institute for Health Research Biomedical Research Centre, Leicester, LE5 4PW UK; 4 MSD UK Limited, London, EC2M 6UR UK; 5 Leicester Diabetes Centre, National Institute for Health Research (NIHR) Applied Research Collaboration - East Midlands (ARC-EM), Leicester, LE5 4PW UK

**Keywords:** Multimorbidity, Cardiometabolic, Type 2 diabetes mellitus, Vascular disease, Coronary heart disease, Cardiovascular disease, Primary health care, Specialist health care, Consultations, Electronic health records, Retrospective study

## Abstract

**Objective:**

To assess trends in primary and specialist care consultation rates and average length of consultation by cardiovascular disease (CVD), type 2 diabetes mellitus (T2DM), or cardiometabolic multimorbidity exposure status.

**Methods:**

Observational, retrospective cohort study used linked Clinical Practice Research Datalink primary care data from 01/01/2000 to 31/12/2018 to assess consultation rates in 141,328 adults with newly diagnosed T2DM, with or without CVD. Patients who entered the study with either a diagnosis of T2DM or CVD and later developed the second condition during the study are classified as the cardiometabolic multimorbidity group. Face to face primary and specialist care consultations, with either a nurse or general practitioner, were assessed over time in subjects with T2DM, CVD, or cardiometabolic multimorbidity. Changes in the average length of consultation in each group were investigated.

**Results:**

696,255 (mean 4.9 years [95% CI, 2.02–7.66]) person years of follow up time, there were 10,221,798 primary and specialist care consultations. The crude rate of primary and specialist care consultations in patients with cardiometabolic multimorbidity (N = 11,881) was 18.5 (95% CI, 18.47–18.55) per person years, 13.5 (13.50, 13.52) in patients with T2DM only (N = 83,094) and 13.2 (13.18, 13.21) in those with CVD (N = 57,974). Patients with cardiometabolic multimorbidity had 28% (IRR 1.28; 95% CI: 1.27, 1.31) more consultations than those with only T2DM. Patients with cardiometabolic multimorbidity had primary care consultation rates decrease by 50.1% compared to a 45.0% decrease in consultations for those with T2DM from 2000 to 2018. Specialist care consultation rates in both groups increased from 2003 to 2018 by 33.3% and 54.4% in patients with cardiometabolic multimorbidity and T2DM, respectively. For patients with T2DM the average consultation duration increased by 36.0%, in patients with CVD it increased by 74.3%, and in those with cardiometabolic multimorbidity it increased by 37.3%.

**Conclusions:**

Annual primary care consultation rates for individuals with T2DM, CVD, or cardiometabolic multimorbidity have fallen since 2000, while specialist care consultations and average consultation length have both increased. Individuals with cardiometabolic multimorbidity have significantly more consultations than individuals with T2DM or CVD alone. Service redesign of health care delivery needs to be considered for people with cardiometabolic multimorbidity to reduce the burden and health care costs.

**Supplementary Information:**

The online version contains supplementary material available at 10.1186/s12933-021-01435-y.

## Introduction

Type 2 diabetes mellitus (T2DM) is a complex metabolic disorder characterized by hyperglycemia resulting from the impairment of insulin secretion or cell tissue resistance to insulin action [1]. Systemic effects of chronically elevated blood glucose render people with T2DM at increased risk of accumulating chronic health conditions over time, a phenomenon termed multimorbidity [2]. In particular, T2DM is strongly associated with cardiovascular diseases, such as coronary heart disease or stroke [3]. Cardiometabolic multimorbidity, the co-occurrence of T2DM and cardiovascular diseases, is rapidly increasing in prevalence worldwide and is a key driver of premature mortality [4, 5] and impaired physical and cognitive function [6]. T2DM is known to have a greater healthcare utilization burden across most health care settings, but this has not been explored in patients with cardiometabolic multimorbidity [7]. Therefore, it is important to understand the relationship between primary and specialist care utilization in patients with cardiometabolic multimorbidity, to help determine future models of healthcare delivery in these patients.

Primary care consultation duration times have increased in recent years, but this has not been investigated in patients with T2DM, cardiovascular disease, and cardiometabolic multimorbidity [8]. Although specialist healthcare utilization has been largely unexplored, there is some evidence to suggest that patients with comorbidities tend to have higher rates of specialist care utilization than their counterparts [9, 10]. Comorbidities in T2DM or cardiovascular diseases has been explored, but there is little research looking at the healthcare use in patients with cardiometabolic multimorbidity [11, 12]. Additionally there are few studies that have directly examined primary and specialist care consultation rates by ethnicity in patients with T2DM and CVD.

In this study we aimed to address these research gaps by quantifying the annual primary and specialist care consultation rates and consultation duration in patients with T2DM, cardiovascular disease or cardiometabolic multimorbidity between 2000 and 2018 in England.

## Methods

This study was approved by the Independent Scientific Advisory Committee of CPRD (protocol number 19_271).

### Study population

The Clinical Practice Research Datalink (CPRD) includes anonymised primary care electronic healthcare records for over 11.3 million UK patients, accounting for 6.9% of the UK population. CPRD is linked to Hospital Episode Statistics (HES) for patients in England, which have been included in this analysis [13]. This retrospective cohort comprised adults aged 18 years or older who were registered with a general practitioner (GP) practice between January 1, 2000 and December 31, 2018, and had a first recorded diagnosis of either T2DM or cardiovascular disease (CVD), defined as coronary heart disease/stroke, in the study period. Additional file 1: Figure S1 shows the study flow diagram for inclusion and exclusion criteria. For more CPRD information the following paper by Herret, et al. can be referred to [13].

### Exposures

The index date was set as the date of the first recorded diagnosis of T2DM (see Additional file 1: Table S1 for medcodes) and/or CVD (Additional file 1: Table S2, S3 for medcodes) within the study duration. Patients were excluded if they had a previous diagnosis of severe CKD (Additional file 1, Table S4 for medcodes) or T2DM or CVD before the study start date. Patients diagnosed with severe CKD before index date, defined as an eGFR less than 30 and dialysis medcodes were excluded (Additional file 1: Table S4 for medcodes). Other exclusion criteria were a diagnosis of cancer before the index date or during the course of the study), coded type 1 diabetes, or gestational diabetes before the index date. Participants were then categorized into three groups: T2DM only, CVD only, and cardiometabolic multimorbidity (T2DM and CVD). Participants belonging to the first two groups (T2DM only or CVD only) who developed during the study period incident CVD or T2DM, respectively, changed their exposed status to cardiometabolic multimorbidity on the date the code related to the incident condition was first recorded. Patients were followed from index date until date of last HES/ONS linkage, date of transition to cardiometabolic multimorbidity (if applicable), death, transfer out of practice date, last data collection date, or the end of the study on 31.12.2018, whichever came first.

Demographic data and laboratory measurements were obtained using CPRD and HES records. Ethnicity was derived from HES admitted patient care records and if it was not available then CPRD patient files were used. Ethnicity groups were defined as the following: White ethnic group, South Asian ethnic group, Black ethnic group and other ethnic group. Medcodes used to define these groups can be found in Additional file 1: Table S5.

Laboratory measurements were obtained between six months before or after the index date and then again between 3 months before and after the date of transition to the cardiometabolic multimorbidity group.

### Consultations

Primary care consultations are routinely entered into CPRD clinical records with different codes representing associated consultancy type and role of the staff who entered the data into the patient’s electronic health record (such as GPs, nurses, health-care assistants, and administrative staff). In this study, we restricted the analysis of primary care consultations to only face-to-face consultations with GPs and nurses, and excluded telephone contacts or consultations where the patient record was opened by the administrative staff. The number of face-to-face consultations was obtained by analysing relevant codes in the ‘consultancy type’ variable (Additional file 1: Table S6). Similarly, relevant codes for GPs and nurses in the entries for ‘staff role’ were identified according to the definition provided by the Vision clinical software system [14] (Additional file 1: Table S7).

CPRD patient records also include the duration of each consultation, defined by the length of time (in minutes) between opening and closing a patient’s electronic health record in the practice software system. Consultation records are rounded down to nearest whole minutes, hence consultation duration of less than one minute are recorded as zero. Furthermore, some of the consultation records have a very long duration (> 60 min). This may occur if the clinical record software is not closed immediately after patient consultation has ended [8]. For our analysis we converted zero-minute consultations into 0.5 min and truncated > 60 min consultation durations to 60 min, in line with previous literature [8, 15]. Consultation duration information is not available in HES Outpatient data.

In HES, outpatient appointments are recorded when the appointment is scheduled, with information on whether the patient attended the appointment. To define specialist consultations, we used HES outpatient records and limited them to appointments attended by a patient. As HES outpatient data are available from 2003, information was collected from this year until the end of the study. HES outpatient records were used to define specialist care consultations as outpatient appointments tend to be with a specialist in a certain field of medicine.

Primary and specialist care consultation records were included in the study only if they occurred within the follow-up period for each individual patient.

### Statistical analysis

The total and mean number of all primary and specialist care consultations were included in the results, as well as the mean annual rate for each type of consultation. Age was first calculated at the index date and for each year thereafter and then categorised as < 50, 50–60, 60–70, 70–80, > 80 years old. For each year of the study, the total number of primary and specialist consultations was estimated overall and stratified by age category, sex, ethnicity and exposure. Patients alive and registered for the full calendar year contributed one-person year of follow-up; those who died or unregistered from their GP practice within a calendar year contributed follow-up time until date of death, transfer out date, last collection date, or date of last linkage to HES/ONS. ONS data was used to determine date of death. Patients who transitioned to cardiometabolic multimorbidity (T2DM + CVD) group contributed separately to consultations and observation time: therefore, patients with T2DM at index date contributed consultations and patient-time to this exposure group until the diagnosis of CVD (if it occurred), when they began contributing consultations and patient-time to the cardiometabolic multimorbidity group.

Crude primary and specialist care consultation rates, overall and stratified, were calculated as the number of consultations per person year (py). Additionally, excess consultations compared to T2DM (reference group) were estimated with a Poisson regression and reported as incidence rate ratios (IRR) adjusted for age, sex, and ethnicity. Additionally, in order to evaluate the effect of having more than one cardiometabolic comorbidity, we included the number of cardiometabolic comorbidities that a patient had before index diagnosis and then again if they developed cardiometabolic multimorbidity. The number of comorbidities were calculated from the following diseases: atrial fibrillation, heart failure, ischaemic heart disease, peripheral arterial disease, microalbuminuria, and non-traumatic amputations. We also analysed the yearly trend of mean consultation duration for face-to-face (GP and nurse) primary care consultations.

All statistical analyses were performed in R Core Team (2020) and Python 3.8.

## Results

### Study cohort

A total of 141,328 patients met the study inclusion criteria. Among these patients, 83,094 (58.8%) were diagnosed with T2DM, 57,974 (41.0%) were diagnosed with CVD, and a small group of subjects were diagnosed with both morbidities on the same day (N = 260, 0.2%). Over the follow-up period, 11,881 (8.4%) patients transitioned to cardiometabolic multimorbidity status. During the follow-up period, a total of 27,861 (19.7%) patients died, of these 7,678 (5.4%) patients had T2DM, 15,549 (11.0%) patients had CVD, and 4,634 (3.3%) patients had been diagnosed with cardiometabolic multimorbidity. The majority of patients were of White ethnic background in all three groups while the proportion for South Asian ethnic groups varied by exposure group: 5.7%, 1.3%, and 3.5% in T2DM, CVD, and cardiometabolic multimorbidity group, respectively (Table 1). Additionally, the proportion of patients from Black ethnic backgrounds was 2.5%, 0.8%, and 1.2% in T2DM, CVD, and cardiometabolic multimorbidity group, respectively (Table 1).

**Table 1:** Characteristics of the study cohort, by exposure status

Variables	T2DM N (%)	CVD N (%)	T2DM AND CVD* N (%)
Total, n	83,094 (58.8%)	57,974 (41.0%)	11,881 (8.4%)
Average duration, mean years	5.49	4.12	4.03
Age, mean [sd] years	61.03 (13.61)	71.15 (14.52)	69.61 (12.20)
Sex (%)			
Men	43,791 (52.7%)	26,599 (45.9%)	6,933 (58.4%)
Women	39,303 (47.2%)	31,375 (54.1%)	4,948 (41.6%)
Ethnicity (%)			
White	73,182 (88.1%)	54,337 (93.7%)	11,056 (93.1%)
South asian	4,720 (5.7%)	763 (1.3%)	417 (3.5%)
Black	2,048 (2.5%)	484 (0.8%)	141 (1.2%)
Other	505 (0.6%)	108 (0.2%)	38 (0.3%)
Smoking status (%)			
Non-smoker	47,659 (57.4%)	32,034 (55.3%)	4,721 (39.7%)
Ex-smoker	5,024 (6.05%)	4,535 (7.8%)	5,187 (43.7%)
Current smoker	30,113 (36.2%)	20,359 (35.11%)	1,945 (16.4%)
Missing	298 (0.4%)	1,046 (1.8%)	28 (0.2%)
Bmi (kg/m^2^)			
Bmi mean [sd]	31.79 (6.81)	27.03 (5.59)	30.60 (6.11)
18.5–24.9	9,507 (11.4%)	12,059 (20.8%)	1,175 (9.9%)
25.0–29.9	24,318 (26.3%)	12,925 (22.3%)	2,664 (22.4%)
30.0–34.9	22,535 (27.12%)	6,344 (10.9%)	2,312 (19.5%)
35.0–39.9	12,048 (14.5%)	2,035 (3.5%)	1,013 (8.5%)
40.0–44.9	5,293 (6.4%)	580 (1.0%)	343 (2.9%)
Hba1c, mean [sd] (%)	7.85 (2.07)	6.00 (1.19)	7.41 (1.69)
Egfr mean [sd] (ml/min/1.73m^2^)	79.00 (20.47)	68.18 (20.26)	80.32 (34.01)
Blood pressure			
Systolic, mean [sd] (mmhg)	139.72 (18.45)	140.68 (22.23)	136.45 (19.83)
Diastolic, mean [sd] (mmhg)	81.73 (10.83)	79.46 (11.92)	76.82 (11.04)
Uric Acid, mean [sd] (µmol)	345.75 (78.35)	344.61 (81.75)	399.41 (110.00)
Comorbidities (%)			
Ischaemic heart disease	9,677 (11.6%)	10,988 (19.0%)	5,708 (48.0%)
Heart failure	2,686 (3.2%)	3,911 (6.9%)	1,415 (11.9%)
Hypertension	41,811 (50.3%)	29,514 (51.0%)	8,642 (72.7%)
Microalbuminuria	197 (0.2%)	89 (0.2%)	526 (4.4%)
Non-traumatic amputation	467 (0.6%)	548 (1.0%)	167 (1.4%)
Atrial fibrillation	4402 (5.3%)	7,259 (12.7%)	3,701 (31.2%)
Peripheral arterial disease	837 (1.0%)	2,178 (3.8%)	1,227 (10.3%)
Consultations			
Total, n	6,155,602	3,154,091	912,105
Primary care, n	5,108,427	2,482,575	622,770
Specialist care, n	1,047,175	671,516	289,335

There were 260 patients that were diagnosed with T2DM and CVD at the same time upon index date they were included in the analysis as cardiometabolic multimorbidity patients

*These patients transitioned during the study to cardiometabolic multimorbidity. Measurements for those with T2DM + CVD were obtained 3 months before or after the transition date (change of status) for this group

There were 27,928 (19.8%) missing BMI values and 64,254 (45.5%) missing Hba1c values for those with T2DM only and CVD only. There were 4101 (34.5%) missing BMI values and 3372 (28.4%) missing Hba1c values in those that transitioned during the study period

*SD* Standard deviation, *T2DM* Type 2 diabetes mellitus, *CVD* coronary heart disease/stroke

### Total face-to-face consultations (primary and specialist)

During 696,255 person years of follow up (mean follow-up: 4.9 years [95% CI, 2.02–7.66]), there were 10,221,798 primary and specialist care consultations. The overall crude consultation rate was 13.7 (95% CI, 13.73–13.75) per person year. Consultation rate was highest among patients with cardiometabolic multimorbidity, 18.5 (18.47–18.55) per person year, compared to 13.5 (13.50–13.52) in patients with T2DM only and 13.2 (13.18–13.21) consultations per person year in those with cardiovascular disease only (Table 2). When compared to patients with T2DM, those with cardiometabolic multimorbidity had 28% (IRR 1.28; 95% CI, 1.27–1.31) higher consultation rate while those with CVD had 8% (IRR 0.92; 95% CI, 0.91–0.93) decreased consultation rate. (Table 2). Total consultation rate increased by 1.13 (1.13, 1.14) per increase of one cardiometabolic comorbidity.

**Table 2:** Crude rates of primary and specialist consultations, by exposure status

Exposure	n (%)	Rate (95% CI)	IRR (95% CI)
Primary Care Consultations			
T2DM	5,108,427 (62.11%)	11.23 (11.22, 11.24)	Reference
CVD	2,482,575 (29.89%)	10.43 (10.42, 10.44)	0.87 (0.87, 0.88)
T2DM + CVD	622,770 (8.00%)	12.67 (12.64, 12.70)	1.08 (1.07, 1.10)
Total	8,213,772	11.07 (11.06, 11.08)	-
Specialist Care Consultations			
T2DM	1,047,175 (51.21%)	2.54 (2.53, 2.54)	Reference
CVD	671,516 (31.87%)	3.05 (3.03, 3.05)	1.17 (1.14, 1.19)
T2DM + CVD	289,335(16.92%)	6.01 (5.99, 6.03)	2.29 (2.23, 2.36)
Total	2,008,026	2.94 (2.94, 2.95)	-
All Consultations			
T2DM	6,155,602 (59.91%)	13.51 (13.50, 13.52)	Reference
CVD	3,154,091 (30.35%)	13.19 (13.18, 13.21)	0.92 (0.91, 0.93)
T2DM + CVD	912,105 (9.74%)	18.51 (18.47, 18.55)	1.28 (1.27, 1.31)
Total	10,221,798	13.74 (13.73, 13.75)	-

*IRR* Incidence rate ratio, *CI* confidence interval

IRR has been adjusted for age, sex, and ethnicity. Rate is number of consultations per person per year

N = 140,165 patients in IRR analyses

Overall consultation rates were higher in females, older age groups, and White ethnic groups (Table 3). In those with T2DM alone the overall consultation rate was 14.5 (14.48–14.52) in females compared to 12.6 (12.60–12.62) in males. In females with CVD alone the overall consultation rate was 14.0 (13.97–14.01) and in males it was 12.32 (12.30–12.34). In the cardiometabolic multimorbidity group, females had an overall consultation rate of 19.8 (19.72–19.85) and rate for males was 17.7 (17.63–17.72). The consultation rate for those aged less than 50 years with T2DM was 12.8 (95% CI, 12.79–12.83) per person year, compared to 10.65 (10.61–10.69) in patients with CVD and 16.5 (16.38–16.65) in patients with cardiometabolic multimorbidity. In patients aged over 80 years old, the crude rates were 15.3 (15.28–15.37), 14.9 (14.90–14.97) and 21.9 (21.75–21.96), in T2DM, CVD and cardiometabolic multimorbidity, respectively. Patients of White and South Asian ethnicity had the highest crude consultation rates, except in the cardiometabolic multimorbidity group, for which patients of Black ethnicity had the highest crude consultation rate (Table 3).

**Table 3:** Crude rates of primary and specialist care consultations by exposure status, stratified by age, sex, and ethnicity

Variables	T2DM			CVD			T2DM + CVD		
	n	Rate (95% CI)	%	n	Rate (95% CI)	%	n	Rate (95% CI)	%
Primary care consultations									
Gender									
Male	2,500,073	10.45 (10.44, 10.46)	48.94%	1,091,357	9.61 (9.60, 9.63)	43.96%	360,637	12.11 (12.07, 12.16)	57.91%
Female	2,608,354	12.10 (12.09, 12.11)	51.06%	1,391,218	11.17 (11.15, 11.19)	56.04%	262,133	13.52 (13.47, 13.57)	42.09%
Age group									
< 50 years	1,038,501	10.62 (10.60, 10.65)	20.33%	194,161	8.02 (7.99, 8.06)	7.82%	39,012	11.37 (11.26, 11.49)	6.26%
50–60 years	1,267,898	10.51 (10.49, 10.53)	24.82%	327,700	8.62 (8.59, 8.65)	13.20%	105,343	11.45 (11.38, 11.52)	16.92%
60–70 years	1,403,264	11.31 (11.29, 11.33)	27.47%	562,928	9.81 (9.78, 9.84)	22.68%	174,775	12.05 (12.00, 12.11)	28.06%
70–80 years	1,026,117	12.28 (12.25, 12.30)	20.09%	748,583	11.35 (11.32, 11.38)	30.15%	195,580	13.57 (13.51, 13.63)	31.40%
> 80 years	372,647	12.95 (12.90, 12.99)	7.29%	649,203	12.37 (12.34, 12.40)	26.15%	108,060	14.19 (14.10, 14.27)	17.35%
Ethnicity									
White	4,621,831	11.37 (11.35, 11.38)	90.47%	2,382,597	10.49 (10.47, 10.50)	95.97%	586,051	12.73 (12.70, 12.77)	94.10%
Black	92,931	9.93 (9.87, 9.99)	1.82%	14,198	8.43 (8.29, 8.57)	0.57%	6,612	12.14 (11.85, 12.43)	1.06%
South asian	262,579	10.94 (10.90, 10.98)	5.14%	30,593	10.19 (10.08, 10.31)	1.23%	21,923	12.47 (12.30, 12.63)	3.52%
Other	19,824	8.29 (8.18, 8.41)	0.39%	3,242	8.01 (7.74, 8.29)	0.13%	1,588	8.92 (8.49, 9.37)	0.25%
Unknown	111,262	8.95 (8.90, 9.00)	2.18%	51,945	9.07 (8.99, 9.15)	2.09%	6,596	10.05 (9.81, 10.30)	1.06%
Total	5,108,427	11.23 (11.22, 11.24)	-	2,482,575	10.43 (10.42, 10.44)	-	622,770	12.67 (12.64, 12.70)	-
Specialist care consultations									
Gender									
Male	523,094	2.44 (2.43, 2.44)	49.95%	313,833	2.96 (2.95, 2.97)	46.73%	166,577	5.72 (5.69, 5.74)	57.57%
Female	524,081	2.65 (2.64, 2.65)	50.05%	357,683	3.12 (3.11, 3.13)	53.27%	122,758	6.47 (6.43, 6.51)	42.43%
Age group									
< 50 years	215,033	2.46 (2.45, 2.47)	20.53%	64,991	2.85 (2.82, 2.87)	9.68%	17,664	5.29 (5.21, 5.37)	6.11%
50–60 years	253,765	2.34 (2.33, 2.35)	24.23%	100,709	2.82 (2.80, 2.83)	15.00%	44,226	4.91 (4.86, 4.95)	15.29%
60–70 years	292,228	2.57 (2.56, 2.58)	27.91%	164,845	3.02 (3.00, 3.03)	24.55%	80,088	5.64 (5.60, 5.68)	27.68%
70–80 years	215,369	2.78 (2.77, 2.79)	20.57%	202,075	3.25 (3.23, 3.26)	30.09%	88,165	6.22 (6.18, 6.27)	30.47%
> 80 years	70,780	2.75 (2.73, 2.77)	6.76%	138,896	3.09 (3.07, 3.10)	20.68%	59,192	8.02 (7.95, 8.08)	20.46%
Ethnicity									
White	950,693	2.56 (2.56, 2.57)	90.79%	647,562	3.06 (3.05, 3.07)	96.43%	269,512	5.98 (5.96, 6.00)	93.15%
Black	24,494	2.92 (2.88, 2.95)	2.34%	5,980	3.84 (3.74, 3.94)	0.89%	4,929	9.06 (8.81, 9.31)	1.70%
South asian	54,487	2.55 (2.52, 2.57)	5.20%	10,328	3.61 (3.54, 3.68)	1.54%	12,176	7.00 (6.87, 7.12)	4.21%
Other	5,377	2.56 (2.49, 2.63)	0.51%	1,021	2.82 (2.65, 2.99)	0.15%	875	4.94 (4.62, 5.28)	0.30%
Unknown	12,124	1.29 (1.27,1.32)	1.16%	6,625	1.67 (1.62, 1.70)	0.99%	1,843	3.29 (3.14, 3.44)	0.64%
Total	1,047,175	2.54 (2.53, 2.54)	-	671,516	3.05 (3.03, 3.05)	–	289,335	6.01 (5.99, 6.03)	–
All consultations									
Gender									
Male	3,023,167	12.61 (12.60, 12.62)	49.11%	1,405,190	12.32 (12.30, 12.34)	44.55%	527,214	17.68 (17.63, 17.72)	57.80%
Female	3,132,435	14.50 (14.48, 14.52)	50.89%	1,748,901	13.99 (13.97, 14.01)	55.45%	384,891	19.79 (19.72, 19.85)	42.20%
Age group									
< 50 years	1,253,534	12.81 (12.79, 12.83)	20.36%	259,152	10.65 (10.61, 10.69)	8.22%	56,676	16.51 (16.38, 16.65)	6.21%
50–60 years	1,521,663	12.59 (12.57, 12.61)	24.72%	428,409	11.23 (11.19, 11.26)	13.58%	149,569	16.24 (16.15, 16.32)	16.40%
60–70 years	1,695,492	13.64 (13.62, 13.66)	27.54%	727,773	12.64 (12.62, 12.67)	23.07%	254,863	17.54 (17.47, 17.61)	27.94%
70–80 years	1,241,486	14.81 (14.79, 14.84)	20.17%	950,658	14.36 (14.33, 14.39)	30.14%	283,745	19.64 (19.56, 19.71)	31.11%
> 80 years	443,427	15.33 (15.28, 15.37)	7.20%	788,099	14.93 (14.90, 14.97)	24.99%	167,252	21.85 (21.75, 21.96)	18.34%
Ethnicity									
White	5,572,524	13.68 (13.67, 13.69)	90.53%	3,030,159	13.28 (13.27, 13.30)	96.07%	855,563	18.55 (18.51, 18.59)	93.80%
Black	117,425	12.50 (12.43, 12.57)	1.91%	20,178	11.87 (11.70, 12.03)	0.64%	11,541	21.01 (20.63, 21.39)	1.27%
South asian	317,066	13.17 (13.12, 13.21)	5.15%	40,921	13.50 (13.37, 13.63)	1.30%	34,099	19.39 (19.18, 19.60)	3.74%
Other	25,201	10.53 (10.40, 10.66)	0.41%	4,263	10.46 (10.15, 10.78)	0.14%	2,463	13.83 (13.30, 14.39)	0.27%
Unknown	123,386	9.89 (9.83, 9.94)	2.00%	58,570	10.12 (10.04, 10.21)	1.86%	8,439	12.76 (12.49, 13.03)	0.93%
Total	6,155,602	13.51 (13.50, 13.52)	–	3,154,091	13.19 (13.18, 13.21)	–	912,105	18.51 (18.47, 18.55)	–

### Primary care consultations

There were a total of 8,213,772 primary care consultations in CPRD. The overall crude rate of primary care consultations was 11.1 (95% CI, 11.06–11.08) per person year; crude rates were 11.2 (11.22–11.24), 10.4 (10.42–10.44), and 12.7 (12.64–12.70) per person-year in subjects with T2DM, CVD, and cardiometabolic multimorbidity, respectively (Table 2). Compared to T2DM only, consultation rates were lower in the CVD group (IRR: 0.87; 95% CI: 0.87–0.88) and higher in the cardiometabolic multimorbidity group (IRR: 1.08; 95% CI: 1.07–1.10). The coefficient for number of cardiometabolic comorbidities was 1.11 (1.10, 1.12); meaning that there was an increase of 1.11 (1.10, 1.12) consultations per increase of one additional comorbidity. Rates of primary care consultations were also higher in females, White ethnic groups and South Asian ethnic groups, and older age groups (Table 3).

### Specialist care consultations

Annual rates of specialist care consultations were generally lower than those for primary care. The total number of specialist care consultations in HES was 2,008,026. The overall crude rate was 2.9 (95% CI, 2.94–2.95) consultations per person year. Patients with T2DM had a crude rate of 2.5 (95% CI, 2.53–2.54), those diagnosed with CVD 3.1 (95% CI, 3.03–3.05), and those with cardiometabolic multimorbidity 6.0 (95% CI, 5.99–6.03) (Table 2). Compared to those with just T2DM, specialist consultation rates were higher in the CVD group (IRR: 1.17; 95% CI, 1.14–1.19) and cardiometabolic multimorbidity group (IRR 2.29; 95% CI, 2.23–2.36). In specialist care consultations, there was an increase of 1.22 (1.21, 1.24) in consultation rate per increase of one cardiometabolic condition. As for primary care, specialist care consultation rates were higher in females and older age groups; patient groups with the highest annual rates were of Black ethnicity (Table 3).

### Consultation rates and visit duration over time

**Fig. 1:**
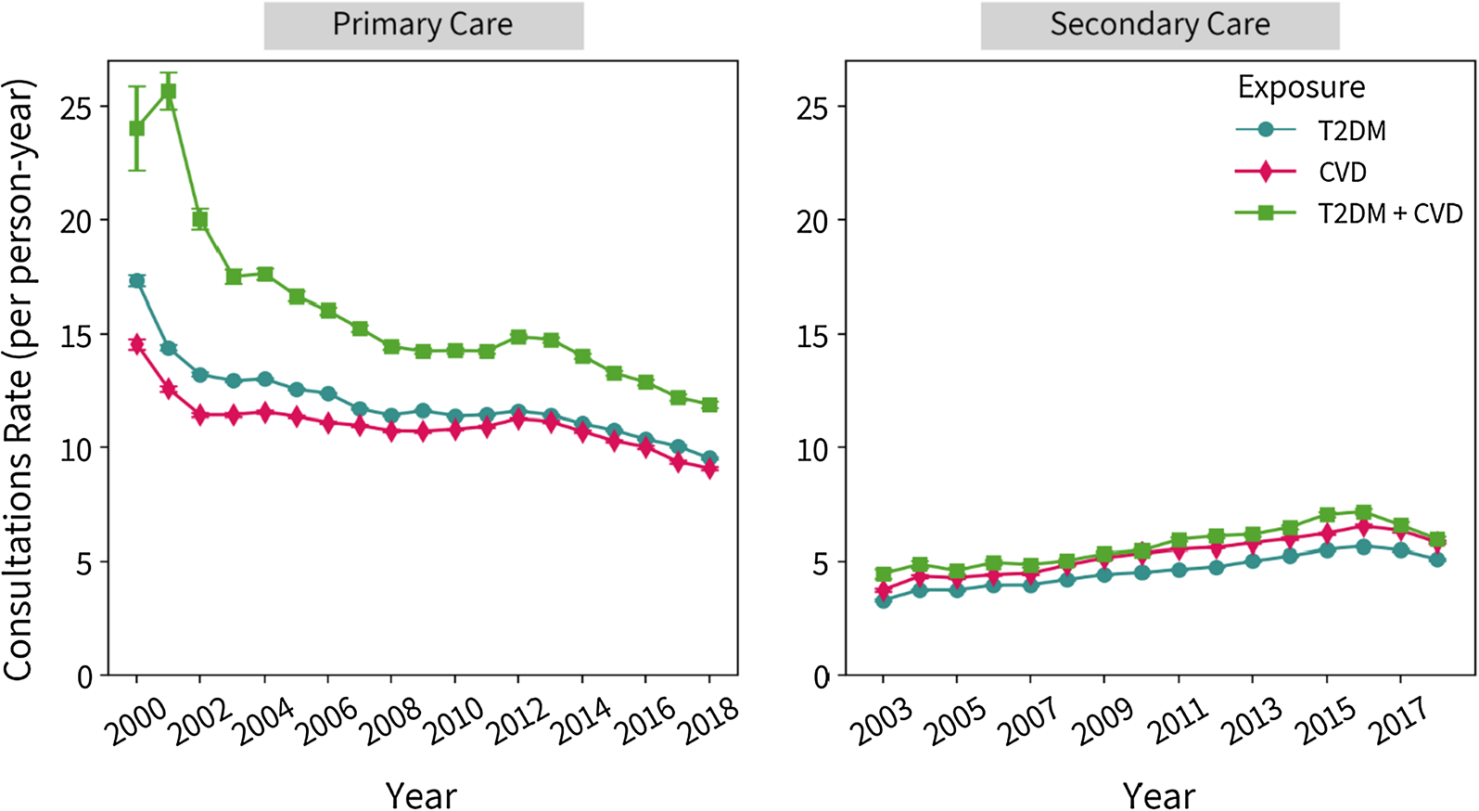
Annual crude consultation rates for primary and specialist care by exposure status. *HES outpatient data was not recorded until 2003, therefore the axes have different time periods

**Fig. 2:**
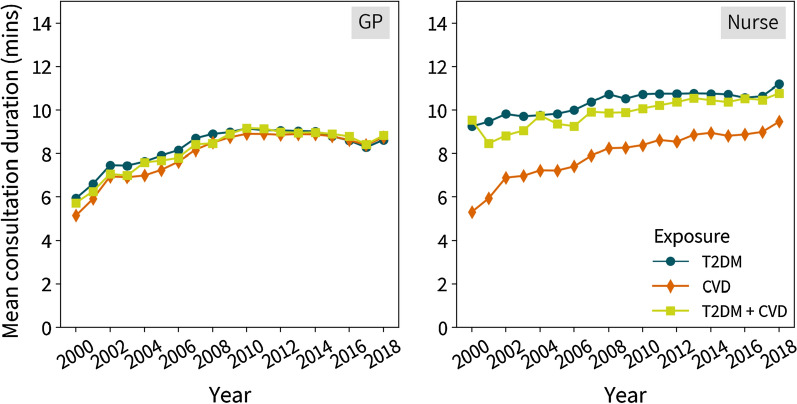
Mean consultation duration for primary care by exposure status and staff role. *Consultation duration is not available for specialist care consultations as HES outpatient data does not include this

Figure 1 shows the annual consultation rates over time by morbidity status, stratified by primary and specialist care. Overall, rates in primary care have decreased since 2000 for all three morbidity groups, and increased in specialist care. Similar patterns were seen across ethnicities, sex and age (Additional file 1: Figure S2–S4). In patients with cardiometabolic multimorbidity primary care consultation rates decreased by 50.1% compared to a 45.0% decrease in consultations for those with T2DM and a 37.5% decrease in CVD patients from 2000 to 2018. Specialist care consultation rates in both groups increased from 2003 to 2018 by 33.3%, 54.8%, and 54.4% in patients with cardiometabolic multimorbidity, cardiovascular disease, and T2DM, respectively. Figure 2 shows the average duration of face-to-face consultation by exposure groups, stratified by GP and nurse consultations. Additional results for annual consultation rates and mean duration stratified by ethnicity, sex and age group are shown in Additional file 1: Figure S2–S7.

The mean duration of all face-to-face consultations (with GPs and nurses) in 2000 was highest in T2DM patients and lowest in those with CVD. For T2DM patients, the average duration increased from 7.2 (SD 9.68) minutes in 2000 to 9.8 (SD 9.87) in 2018, whereas in patients with CVD, increased from 5.2 (SD 7.92) minutes in 2000 to 9.0 (SD 9.39) in 2018 (Fig. 2), indicating a 36.0% and 74.3% increase, respectively. In patients with cardiometabolic multimorbidity, the mean duration increased by 37.3% duration the study duration, from 7.0 (SD 10.36) minutes in 2000 to 9.7 (SD 10.02) in 2018.

Mean duration of face-to-face consultations with nurses were consistently higher over the study period than those with GPs for patients with T2DM and cardiac multimorbidity. The average duration of GP consultations with T2DM and cardiometabolic multimorbidity patients were respectively 5.9 (SD 7.84) and 5.7 (SD 8.38) minutes in 2000, and increased by 45% and 54%, respectively, in 2018. Corresponding results for nurse consultations were respectively 9.24 (SD 11.80) and 9.53 (SD 13.03) minutes in 2000, which rose by 21% and 13%, respectively, in 2018. In patients with CVD, the average GP and nurse consultation time increased by 70% and 79%, respectively, during the study time.

## Discussion

This study highlights the increased burden of cardiometabolic multimorbidity on healthcare utilisation in both primary and specialist care in England. We found that patients with both T2DM and CVD had higher annual consultation rates, compared to patients with T2DM or CVD alone. Overall consultation rates in individuals with cardiometabolic multimorbidity was around 19 visits per patient per year, highlighting a significant burden of healthcare delivery in this group. Additionally, consultation rates in primary care were higher than in specialist care in all three groups, fitting with the structure of diabetes care in England. In general, it appears that patients without chronic conditions have fewer consultations than patients with one or more chronic conditions [16]. Currently the healthcare framework in many developed countries is based on treating single-diseases individually, with many GPs limiting their consultations to a discussion of one health problem [17]. This may not be the best model of care for individuals with multimorbidity.

Our results also show that patients from White and South Asian ethnic groups (see Additional file 1: Table S5 for codes) were more likely to present for a T2DM consultation than CVD in specialist consultations compared to patients from Black ethnic groups (see Additional file 1: Table S5 for codes). This could suggest that patients from Black ethnic groups are waiting to present acutely with CVD compared to patients from White and South Asian ethnic groups or that the current healthcare CVD prevention strategies are not adequately engaging this group of patients. Overall, consultations were significantly lower in patients with CVD compared to T2DM patients in primary care than in specialist care. This may be due to patients with CVD presenting acutely in specialist consultations than T2DM patients, whom are typically diagnosed and followed up in primary care. These results suggest that earlier screening programmes may be helpful in preventing acute CVD cases in all ethnicities.

Although this is the first analysis assessing consultation rates in patients with cardiometabolic multimorbidity, studies have previously found that co-morbidities in patients with T2DM or CVD increase primary care costs, health service use, and have higher rates of hospitalisation [18–20]. In this study we found that there was a decrease in overall primary care visits from 2000 to 2018, although consultation rates were the highest in the cardiometabolic multimorbidity exposure group. A study using consultation data from Norwegian clinical practices found that continuity of care was high in patients with chronic disease, it would be beneficial to investigate continuity of care in patients with comorbidities, such as cardiometabolic multimorbidity, in the future [21]. Additionally, in line with our results, a study by Ganguli et al. found that amongst commercially insured adults in the United States, primary care visits have decreased by 24% between 2008 and 2016 [22].

Our analysis also shows that the average duration of consultation for patients in all three groups increased consistently over time, indicating additional clinical load on GPs and practice nurses as found in previous studies [23]. The longer consultation duration with time may be due to increasing number of patients with long-term conditions included in the cohort over time. GP consultation time was similar for the three patient groups, but nurse consultations showed higher consultation length for patients with T2DM and cardiac multimorbidity. As we did not include prior comorbidities and practice characteristics, further work in this regard may reflect how these factors also affect the length of consultation [24].

This is one of the first studies to evaluate specialist care consultations by multimorbidity status. Our study shows that although the absolute number of specialist care consultations is less than those seen in primary care settings, the impact of cardiometabolic multimorbidity (6.0%, 95% CI: 5.99–6.03) on specialist care services is relatively high as the crude rate of specialist care consultations in this patient group is double the rate of either patients with T2DM (2.5%, 95% CI: 2.53–2.54) or CVD (3.1%, 95% CI: 3.03–3.05). As we were not able to evaluate the consultation duration in secondary care, this needs to be further explored to fully gauge the impact cardiometabolic multimorbidity patients have on specialist/hospital settings.

The use of a large cohort of individuals with a representative coverage of the population of England is a major strength of this study. Overall, primary and specialist care visits are well recorded in both CPRD and HES; therefore, our findings are representative for the UK population with respect to age, sex, and ethnicity [13]. The large cohort also allowed us to estimate rates in clinically relevant subgroups defined by age, sex, and ethnicity.

There are some limitations in this study. In some cases, the duration of the consultation was not adequately recorded, with reported times of 0 s or greater than an hour, and assumptions had to be made; furthermore, 3.5% of patients were missing data on ethnicity. Our definition of cardiometabolic multimorbidity was limited to T2DM and cardiovascular disease (defined as coronary heart disease or stroke), cardiometabolic multimorbidity can also include other diseases such as hypertension, congestive cardiac failure and CKD that often co-exist with T2DM and CVD [25, 26]. Further we could not identify insulin resistance or prediabetes in those with CVD [27]. Additionally, it is not possible to gather specific data regarding coronary vessels anatomy or ejection fraction from CPRD, which draws data from primary care services. The management of these additional diseases, insulin resistance, and other risk factors mentioned above, can prolong consultation times and are likely to further increase consultation rates [25, 27, 28]. Additionally, the specialist care data used to define was crude and could not be broken down by speciality. The consultation duration for secondary care consultations was not available in the HES datasets. Furthermore, we did not set out to address financial burden in this paper, although Coles et al. found that those with T2DM for each additional comorbidity the mean cost of face-to-face consultations increased [29].

This study covers an extensive analysis of CPRD and highlighted the increased burden of cardiometabolic multimorbidity on primary and specialist care resources in England. Our results support that patients with cardiometabolic multimorbidity have an increased rate of both primary and specialist care consultations compared to patients with either just T2DM or CVD. As medical specialist training and clinical care is largely guided by the presence of one disease therefore healthcare workers have little experience in how to care for patients with comorbidities [30]. In many developed countries, most conditions are managed through primary care in appointments lasting just 10–15 min regardless of the number of conditions a patient presents with. Therefore, it is unlikely these appointments lend enough time to deal with complex health issues that arise in comorbid patients [21]. As cardiometabolic multimorbidity is predicted to increase in coming years, it is essential to ensure that access to healthcare is structured in an efficient and cost-effective manner for this patient group.

Additionally, the results of this study support that different ethnicity groups appear with cardiometabolic events at varying times of disease progression, such as White and South Asian ethnic groups have higher rates of primary care consultations and Black ethnic groups have higher rates of specialist care consultations in patients with T2DM. Our study demonstrates the importance of adopting a holistic approach, taking ethnic differences into consideration, in primary and specialist care for patients transitioning from one major chronic condition to developing another lifelong comorbidity. Future studies are needed to gauge why different ethnic groups present at varying times in disease progression so that public health programmes can be created to accurately surveillance these patients.

Overall our study highlights the burden those with cardiometabolic multimorbidity experience in England’s healthcare system. Different pathways of patient care need to be considered for these patients that would result in more efficient and economical healthcare visits, while. Future studies are needed to assess the costs associated with increased consultations and duration times for England’s healthcare system in order to fully understand the burden on not only patients but also the National Health Service.

## Supplementary Information

**Additional file 1.** This file includes additional tables and graphs that further the results of this study. Medcodes and ICD-10 code lists that were used to define variables, outcomes, and exposures in this study have also been included in this file.

## Data Availability

The data that support the findings of this study are available from CPRD but restrictions apply to the availability of these data, which were used under license for the current study, and so are not publicly available. Data are however available from the authors upon reasonable request and with permission of CPRD.
