# Supplementary material for: Consultation rates in people with type 2 diabetes with and without vascular complications: a retrospective analysis of 141,328 adults in England
Source: Cardiovasc Diabetol. 2022 Jan 10;21:8. doi: 10.1186/s12933-021-01435-y (PMC8744247; doi:10.1186/s12933-021-01435-y)

**Supplementary Material**

Consultation rates in people with type 2 diabetes with and without vascular complications: A retrospective analysis of 141,328 adults in England

**Sophia Abner,** Statistician/Epidemiologist, Leicester Real World Evidence Unit, Diabetes Research Centre, Leicester General Hospital, University of Leicester, Leicester, LE5 4PW, UK

**Clare L Gillies,** Lecturer in Medical Statistics, Leicester Real World Evidence Unit, Diabetes Research Centre, Leicester General Hospital, University of Leicester, Leicester, LE5 4PW, UK

**Sharmin Shabnam,** Data Scientist, Leicester Real World Evidence Unit, Diabetes Research Centre, Leicester General Hospital, University of Leicester, Leicester, LE5 4PW, UK

**Francesco Zaccardi**, Clinical Epidemiologist, Leicester Real World Evidence Unit, Diabetes Research Centre, Leicester General Hospital, University of Leicester, Leicester, LE5 4PW, UK

**Samuel Seidu**, Clinical Lecturer in Primary Care, **,** Leicester Diabetes Centre, Leicester General Hospital, Leicester, LE5 4PW, UK

**Melanie J Davies**, Professor of Diabetes Medicine, National Institute for Health Research Biomedical Research Centre, Leicester Diabetes Centre, Leicester, LE5 4PW, UK

**Tobi Adeyemi,** MSD UK Limited, London, EC2M 6UR, UK

**Kamlesh Khunti*,** Professor of Primary Care Diabetes and Vascular Medicine, National Institute for Health Research (NIHR) Applied Research Collaboration - East Midlands (ARC-EM), Leicester Diabetes Centre, Leicester, LE5 4PW, UK

**David Webb*,** Senior Lecturer in Diabetes Medicine**,** Leicester Diabetes Centre, Leicester General Hospital, Leicester, LE5 4PW, UK

### Table S1. Type 2 Diabetes - Medical codes (CPRD) and ICD-10 codes (HES)

| ICD Codes | Description |
| --- | --- |
| E11.xx | Non-insulin dependent diabetes |
| O24.1 | Pre-existing diabetes mellitus, non-insulin-dependent |

| Medcode | Description |
| --- | --- |
| 64668 | Insulin treated Type II diabetes mellitus |
| 106061 | Type II diabetes mellitus with ketoacidotic coma |
| 101801 | Type II diabetic dietary review |
| 36633 | Hyperosmolar non-ketotic state in type 2 diabetes mellitus |
| 28769 | Diabetic on insulin and oral treatment |
| 17262 | Non-insulin-dependent diabetes mellitus with retinopathy |
| 62674 | Type 2 diabetes mellitus with mononeuropathy |
| 61071 | Type 2 diabetes mellitus with hypoglycaemic coma |
| 26054 | Type 2 diabetes mellitus with persistent proteinuria |
| 39317 | Diabetes mellitus, adult onset, + neurological manifestation |
| 108005 | Type 2 diabetes mellitus with multiple complications |
| 98616 | Type II diabetes mellitus with neurological complications |
| 35385 | Type 2 diabetes mellitus with neuropathic arthropathy |
| 40401 | Non-insulin dependent diabetes mellitus with gangrene |
| 111798 | Type II diabetes mellitus with exudative maculopathy |
| 49074 | Type 2 diabetes mellitus with ulcer |
| 1407 | Insulin treated Type 2 diabetes mellitus |
| 63357 | Diabetes mellitus, adult, + peripheral circulatory disorder |
| 25041 | Dietary advice for type II diabetes |
| 18278 | Insulin treated Type 2 diabetes mellitus |
| 50527 | Type II diabetes mellitus with polyneuropathy |
| 107701 | Hyperosmolar non-ketotic state in type II diabetes mellitus |
| 37806 | Type 2 diabetes mellitus with peripheral angiopathy |
| 758 | Type 2 diabetes mellitus |
| 36695 | Diabetes mellitus autosomal dominant type 2 |
| 24693 | Non-insulin dependent diabetes mellitus with arthropathy |
| 51756 | Type 2 diabetes mellitus with ketoacidotic coma |
| 69278 | Non-insulin depend diabetes mellitus with diabetic cataract |
| 18390 | Type 2 diabetes mellitus with persistent microalbuminuria |
| 58604 | Type II diabetes mellitus with retinopathy |
| 42762 | Type 2 diabetes mellitus with retinopathy |
| 95351 | Type II diabetes mellitus with mononeuropathy |
| 104639 | Type II diabetes mellitus with peripheral angiopathy |
| 56268 | Type II diabetes mellitus with hypoglycaemic coma |
| 34268 | Type 2 diabetes mellitus with neurological complications |
| 34912 | Non-insulin dependent diabetes mellitus with ulcer |

| 25591 | Type 2 diabetes mellitus with exudative maculopathy |
| --- | --- |
| 47954 | Type 2 diabetes mellitus without complication |
| 107824 | Type II diabetes mellitus in remission |
| 98723 | Type II diabetes mellitus with hypoglycaemic coma |
| 59253 | Type 2 diabetes mellitus with arthropathy |
| 34450 | Hyperosmolar non-ketotic state in type 2 diabetes mellitus |
| 37648 | Insulin treated non-insulin dependent diabetes mellitus |
| 18264 | Insulin treated Type II diabetes mellitus |
| 48192 | Type II diabetes mellitus with diabetic cataract |
| 70316 | Type 2 diabetes mellitus with ophthalmic complications |
| 59725 | Type II diabetes mellitus with ophthalmic complications |
| 57278 | Type II diabetes mellitus with renal complications |
| 64571 | Type II diabetes mellitus with nephropathy |
| 24836 | Type 2 diabetes mellitus with nephropathy |
| 63762 | Diabetes mellitus, adult onset, + unspecified complication |
| 50609 | Pre-existing diabetes mellitus, non-insulin-dependent |
| 55842 | Non-insulin-dependent diabetes mellitus with neuro comps |
| 65267 | Type 2 diabetes mellitus with multiple complications |
| 35105 | Diabetes mellitus, adult onset, with renal manifestation |
| 41389 | Diabetes mellitus, adult onset, + ophthalmic manifestation |
| 62146 | Non-insulin-dependent diabetes mellitus with multiple comps |
| 65704 | Type 2 diabetes mellitus with ulcer |
| 55075 | Type II diabetes mellitus with ulcer |
| 104323 | Type II diabetes mellitus with gangrene |
| 53392 | Type II diabetes mellitus without complication |
| 110611 | Type 2 diabetes mellitus in remission |
| 46917 | Type 2 diabetes mellitus with hypoglycaemic coma |
| 66965 | Type 2 diabetes mellitus with neuropathic arthropathy |
| 43139 | Diabetes mellitus, adult onset, with hyperosmolar coma |
| 12640 | Type 2 diabetes mellitus with nephropathy |
| 44779 | Type 2 diabetes mellitus with diabetic cataract |
| 1684 | Diabetic on oral treatment |
| 103902 | Type II diabetes mellitus with arthropathy |
| 54899 | Type II diabetes mellitus with peripheral angiopathy |
| 60699 | Type 2 diabetes mellitus with peripheral angiopathy |
| 105784 | Type 2 diabetes mellitus without complication |
| 109103 | Type II diabetes mellitus without complication |
| 50429 | Non-insulin-dependent diabetes mellitus with ophthalm comps |
| 18777 | Type 2 diabetes mellitus with renal complications |
| 59365 | Non-insulin dependent diabetes mellitus with nephropathy |
| 12736 | Type 2 diabetes mellitus with gangrene |
| 47816 | Type II diabetes mellitus with neuropathic arthropathy |
| 67905 | Type II diabetes mellitus with neurological complications |
| 45919 | Type 2 diabetes mellitus with neurological complications |
| 43227 | Type II diabetes mellitus with multiple complications |
| 33807 | Diabetes mellitus, adult with gangrene |
| 110344 | Diabetic on non-insulin injectable medication |
| 49655 | Type II diabetes mellitus with retinopathy |
| 106528 | Type II diabetes mellitus with ketoacidosis |

| 44982 | Type 2 diabetes mellitus with diabetic cataract |
| --- | --- |
| 4513 | Non-insulin dependent diabetes mellitus |
| 47321 | Type 2 diabetes mellitus with ophthalmic complications |
| 24458 | Type II diabetes mellitus - poor control |
| 45913 | Type 2 diabetes mellitus - poor control |
| 50813 | Type II diabetes mellitus with mononeuropathy |
| 102201 | Type II diabetes mellitus with nephropathy |
| 52303 | Non-insulin-dependent diabetes mellitus with renal comps |
| 29979 | Non-insulin-dependent diabetes mellitus without complication |
| 45467 | Non-insulin dependent diabetes mellitus with polyneuropathy |
| 68843 | Diabetes mellitus, adult onset, with ketoacidotic coma |
| 63371 | Diabetes mellitus, adult, + other specified manifestation |
| 14803 | Diabetes mellitus, adult onset, no mention of complication |
| 18496 | Type 2 diabetes mellitus with retinopathy |
| 50225 | Type II diabetes mellitus with renal complications |
| 32627 | Type 2 diabetes mellitus with ketoacidosis |
| 102611 | Type 2 diabetic dietary review |
| 93727 | Type II diabetes mellitus with diabetic cataract |
| 5884 | NIDDM - Non-insulin dependent diabetes mellitus |
| 17859 | Type 2 diabetes mellitus |
| 47315 | Type II diabetes mellitus - poor control |
| 100964 | Type II diabetes mellitus with ophthalmic complications |
| 43785 | Non-insulin dependent diabetes mellitus with hypoglyca coma |
| 60796 | Type II diabetes mellitus with persistent proteinuria |
| 8403 | Non-insulin dependent diabetes mellitus - poor control |
| 72320 | Non-insulin dependent diabetes mellitus with mononeuropathy |
| 109197 | Type II diabetes mellitus with neuropathic arthropathy |
| 103597 | Family history of diabetes mellitus type II |
| 54856 | Diabetes mellitus, adult onset, with ketoacidosis |
| 63690 | Type 2 diabetes mellitus with gastroparesis |
| 18209 | Type 2 diabetes mellitus with renal complications |
| 47409 | Type II diabetes mellitus with polyneuropathy |
| 109865 | Type 2 diabetes mellitus with polyneuropathy |
| 14889 | Maturity onset diabetes |
| 46150 | Type 2 diabetes mellitus with gangrene |
| 62107 | Type II diabetes mellitus with gangrene |
| 91646 | Type II diabetes mellitus with ulcer |
| 25627 | Type 2 diabetes mellitus - poor control |
| 18219 | Type II diabetes mellitus |
| 18143 | Type II diabetes mellitus with arthropathy |
| 506 | Non-insulin dependent diabetes mellitus |
| 22884 | Type II diabetes mellitus |
| 59991 | Maturity onset diabetes in youth type 2 |
| 49869 | Type 2 diabetes mellitus with arthropathy |

###

### Table S2. Coronary heart disease/Myocardial infarction - Medical codes (CPRD) and ICD-10 codes (HES)

| ICD-10 | Description |
| --- | --- |
| I21 | Acute myocardial infarction |
| I22 | Subsequent myocardial infarction |
| I23 | Certain current complications following acute myocardial infarction |
| I24 | Other acute ischaemic heart diseases |

| Medcode | Description |
| --- | --- |
| Myocardial Infarction | |
| 241 | acute myocardial infarction |
| 1677 | mi - acute myocardial infarction |
| 1678 | inferior myocardial infarction nos |
| 3704 | acute subendocardial infarction |
| 4017 | old myocardial infarction |
| 5387 | other specified anterior myocardial infarction |
| 7783 | ecg: myocardial infarction |
| 8935 | acute inferolateral infarction |
| 9413 | other acute and subacute ischaemic heart disease |
| 9507 | acute non-q wave infarction |
| 10562 | acute non-st segment elevation myocardial infarction |
| 12139 | acute anterolateral infarction |
| 12229 | acute st segment elevation myocardial infarction |
| 12743 | revascularisation of wall of heart |
| 14658 | acute myocardial infarction nos |
| 14897 | anterior myocardial infarction nos |
| 14898 | lateral myocardial infarction nos |
| 16408 | healed myocardial infarction |
| 17464 | personal history of myocardial infarction |
| 17689 | silent myocardial infarction |
| 17872 | acute anteroseptal infarction |
| 18842 | subsequent myocardial infarction |
| 23579 | postmyocardial infarction syndrome |
| 23892 | posterior myocardial infarction nos |
| 27951 | other acute and subacute ischaemic heart disease |
| 28736 | acute atrial infarction |
| 29643 | acute inferoposterior infarction |
| 29758 | acute transmural myocardial infarction of unspecif site |
| 30421 | cardiac rupture following myocardial infarction (mi) |
| 32272 | postoperative myocardial infarction |
| 32854 | acute posterolateral myocardial infarction |
| 34803 | other acute myocardial infarction |

| 35119 | post infarction pericarditis |
| --- | --- |
| 35674 | h/o: myocardial infarct <60 |
| 36423 | certain current complication follow acute myocardial infarct |
| 38609 | subsequent myocardial infarction of inferior wall |
| 39904 | ecg: old myocardial infarction |
| 40399 | h/o: myocardial infarct >60 |
| 40429 | acute anteroapical infarction |
| 41221 | acute septal infarction |
| 45809 | subsequent myocardial infarction of anterior wall |
| 46017 | other acute myocardial infarction nos |
| 46112 | postoperative transmural myocardial infarction anterior wall |
| 46166 | subsequent myocardial infarction of unspecified site |
| 46276 | postoperative transmural myocardial infarction inferior wall |
| 50372 | h/o: myocardial infarction in last year |
| 52705 | ecg: lateral infarction |
| 59032 | ecg: myocardial infarct nos |
| 61670 | diab mellit insulin-glucose infus acute myocardial infarct |
| 62626 | acute papillary muscle infarction |
| 63467 | true posterior myocardial infarction |
| 68401 | [x]other forms of acute ischaemic heart disease |
| 68748 | postoperative myocardial infarction, unspecified |
| 72562 | subsequent myocardial infarction of other sites |
| 95550 | admit ischaemic heart disease emergency |
| 96838 | [x]acute transmural myocardial infarction of unspecif site |
| 99991 | [x]subsequent myocardial infarction of unspecified site |
| 100139 | history of myocardial infarction |
| 100437 | exception reporting: myocardial infarction quality indicator |
| 102917 | exc myocar infarction quality indicators: patient unsuitable |
| 106812 | postoperative transmural myocardial infarction unspec site |
| 109035 | [x]subsequent myocardial infarction of other sites |

### Table S3. Stroke/Cerebrovascular accidents - Medical codes (CPRD) and ICD-10 codes (HES)

| ICD-10 | | Description |  |
| --- | --- | --- | --- |
|  | Intracranial haemorrhages | | |
| I60 | | Subarachnoid haemorrhage | |
| I60.0 | | Subarachnoid haemorrhage from carotid siphon and bifurcation | |
| I60.1 | | Subarachnoid haemorrhage from middle cerebral artery | |
| I60.2 | | Subarachnoid haemorrhage from anterior communicating artery | |
| I60.3 | | Subarachnoid haemorrhage from posterior communicating artery | |
| I60.4 | | Subarachnoid haemorrhage from basilar artery | |
| I60.5 | | Subarachnoid haemorrhage from vertebral artery | |
| I60.6 | | Subarachnoid haemorrhage from other intracranial arteries | |
| I60.7 | | Subarachnoid haemorrhage from intracranial artery, unspecified | |
| I60.8 | | Other subarachnoid haemorrhage | |
| I60.9 | | Subarachnoid haemorrhage, unspecified | |
| I61 | | Intracerebral haemorrhage | |
| I61.0 | | Intracerebral haemorrhage in hemisphere, subcortical | |
| I61.1 | | Intracerebral haemorrhage in hemisphere, cortical | |
| I61.2 | | Intracerebral haemorrhage in hemisphere, unspecified | |
| I61.3 | | Intracerebral haemorrhage in brain stem | |

| I61.4 | | Intracerebral haemorrhage in cerebellum |
| --- | --- | --- |
| I61.5 | | Intracerebral haemorrhage, intraventricular |
| I61.6 | | Intracerebral haemorrhage, multiple localized |
| I61.8 | | Other intracerebral haemorrhage |
| I61.9 | | Intracerebral haemorrhage, unspecified |
| I62 | | Other nontraumatic intracranial haemorrhage |
| I62.0 | | Subdural haemorrhage (acute)(nontraumatic) |
| I62.1 | | Nontraumatic extradural haemorrhage |
| I62.9 | | Intracranial haemorrhage (nontraumatic), unspecified |
|  | Cerebrovascular diseases | |
| I63 | | Cerebral infarction |
| I63.0 | | Cerebral infarction due to thrombosis of precerebral arteries |
| I63.1 | | Cerebral infarction due to embolism of precerebral arteries |
| I63.2 | | Cerebral infarction due to unspecified occlusion or stenosis of precerebral arteries |
| I63.3 | | Cerebral infarction due to thrombosis of cerebral arteries |
| I63.4 | | Cerebral infarction due to embolism of cerebral arteries |
| I63.5 | | Cerebral infarction due to unspecified occlusion or stenosis of cerebral arteries |
| I63.6 | | Cerebral infarction due to cerebral venous thrombosis, nonpyogenic |
| I63.8 | | Other cerebral infarction |
| I63.9 | | Cerebral infarction, unspecified |
| I64 | | Stroke, not specified as haemorrhage or infarction |
| I65 | | Occlusion and stenosis of precerebral arteries, not resulting in cerebral infarction |
| I65.0 | | Occlusion and stenosis of vertebral artery |
| I65.1 | | Occlusion and stenosis of basilar artery |
| I65.2 | | Occlusion and stenosis of carotid artery |
| I65.3 | | Occlusion and stenosis of multiple and bilateral precerebral arteries |
| I65.8 | | Occlusion and stenosis of other precerebral artery |
| I65.9 | | Occlusion and stenosis of unspecified precerebral artery |
| I66 | | Occlusion and stenosis of cerebral arteries, not resulting in cerebral infarction |
| I66.0 | | Occlusion and stenosis of middle cerebral artery |
| I66.1 | | Occlusion and stenosis of anterior cerebral artery |
| I66.2 | | Occlusion and stenosis of posterior cerebral artery |
| I66.3 | | Occlusion and stenosis of cerebellar arteries |
| I66.4 | | Occlusion and stenosis of multiple and bilateral cerebral arteries |
| I66.8 | | Occlusion and stenosis of other cerebral artery |
| I66.9 | | Occlusion and stenosis of unspecified cerebral artery |
| I67 | | Other cerebrovascular diseases |
| I67.0 | | Dissection of cerebral arteries, nonruptured |
| I67.1 | | Cerebral aneurysm, nonruptured |
| I67.2 | | Cerebral atherosclerosis |
| I67.3 | | Progressive vascular leukoencephalopathy |
| I67.4 | | Hypertensive encephalopathy |
| I67.5 | | Moyamoya disease |
| I67.6 | | Nonpyogenic thrombosis of intracranial venous system |
| I67.7 | | Cerebral arteritis, not elsewhere classified |

| I67.8 | Other specified cerebrovascular diseases |
| --- | --- |
| I67.9 | Cerebrovascular disease, unspecified |
| I68 | Cerebrovascular disorders in diseases classified elsewhere |
| I68.0 | Cerebral amyloid angiopathy |
| I68.1 | Cerebral arteritis in infectious and parasitic diseases classified elsewhere |
| I68.2 | Cerebral arteritis in other diseases classified elsewhere |
| I68.8 | Other cerebrovascular disorders in diseases classified elsewhere |
| I69 | Sequelae of cerebrovascular disease |
| I69.0 | Sequelae of subarachnoid haemorrhage |
| I69.1 | Sequelae of intracerebral haemorrhage |
| I69.2 | Sequelae of other nontraumatic intracranial haemorrhage |
| I69.3 | Sequelae of cerebral infarction |
| I69.4 | Sequelae of stroke, not specified as haemorrhage or infarction |
| I69.8 | Sequelae of other and unspecified cerebrovascular diseases |

| Medcode | Description |
| --- | --- |
| 1433 | transient ischaemic attack |
| 1469 | stroke and cerebrovascular accident unspecified |
| 2418 | cerebrovascular disease |
| 2760 | peripheral vascular disease nos |
| 3149 | cerebral infarction nos |
| 3530 | peripheral vascular disease nos |
| 5141 | congestive cardiomyopathy |
| 5602 | cerebellar infarction |
| 5702 | peripheral ischaemic vascular disease |
| 5871 | h/o: stroke |
| 6116 | cva - cerebrovascular accident unspecified |
| 6228 | sequelae of stroke,not specfd as h'morrhage or infarction |
| 6960 | cva - cerebrovascular accid due to intracerebral haemorrhage |
| 7138 | [v]personal history of cerebrovascular accident (cva) |
| 7535 | primary dilated cardiomyopathy |
| 8443 | brain stem stroke syndrome |
| 9402 | secondary dilated cardiomyopathy |
| 9985 | left sided cerebral infarction |
| 10062 | cerebrovascular disease nos |
| 10444 | h/o: cardiovascular disease |
| 10504 | right sided cerebral infarction |
| 10962 | exception reporting: stroke quality indicators |
| 11039 | excepted from stroke quality indicators: patient unsuitable |
| 11074 | excepted from stroke quality indicators: informed dissent |
| 12555 | generalised ischaemic cerebrovascular disease nos |
| 13577 | other cerebrovascular disease |
| 13707 | stroke / transient ischaemic attack referral |
| 15252 | brainstem infarction nos |
| 16148 | peripheral vascular complications of care |
| 17322 | cerebellar stroke syndrome |
| 17645 | angiocardiography of left side of heart nec |
| 18499 | peripheral vascular disease monitoring |
| 18687 | dna - did not attend stroke clinic |
| 18804 | referral to stroke clinic |
| 19348 | [v]personal history of stroke |
| 22672 | cardiovascular disease, unspecified |
| 23361 | late effects of cerebrovascular disease |
| 24035 | subacute confusional state, of cerebrovascular origin |
| 24446 | cerebral infarction due to embolism of precerebral arteries |
| 25114 | acute confusional state, of cerebrovascular origin |
| 25615 | brainstem infarction |
| 27975 | cerebral infarction due to embolism of cerebral arteries |
| 28753 | stroke/transient ischaemic attack monitoring first letter |
| 28914 | haemorrhagic stroke monitoring |
| 31218 | stroke/transient ischaemic attack monitoring administration |
| 32434 | renal infarction |
| 32959 | seen in stroke clinic |
| 34117 | other cerebrovascular disease os |
| 34135 | h/o: cva/stroke |
| 34245 | stroke/transient ischaemic attack monitoring second letter |
| 34375 | stroke/transient ischaemic attack monitoring third letter |
| 35382 | angiocardiography abnormal |
| 36609 | atherosclerotic cardiovascular disease |
| 36717 | cerebral infarction due to thrombosis of cerebral arteries |
| 37391 | revascularisation for impotence |
| 37493 | other cerebrovascular disease nos |
| 38907 | other specified peripheral vascular disease |
| 39403 | sequelae of cerebral infarction |
| 40053 | generalised ischaemic cerebrovascular disease nos |
| 40098 | suspected cerebrovascular disease |
| 40834 | other primary cardiomyopathy nos |
| 41488 | constrictive cardiomyopathy |
| 41577 | h/o: cerebrovascular disease |
| 41835 | postoperative subendocardial myocardial infarction |
| 42248 | discharge from stroke serv |
| 42335 | cerebrovascular disorders in the puerperium |
| 43001 | dna - did not attend peripheral vascular disease clinic |
| 47607 | cva - cerebrovascular accident in the puerperium |
| 51138 | sequelae/other + unspecified cerebrovascular diseases |
| 51195 | h/o: major vascular surgery |
| 51311 | other specified cerebrovascular disease |
| 51465 | stroke/transient ischaemic attack monitoring verbal invitati |
| 52637 | canadian cardiovascular society classification of angina |
| 53745 | [x]other cerebral infarction |
| 54744 | cerebral degeneration due to cerebrovascular disease |
| 55351 | delivery of rehabilitation for stroke |
| 56458 | ref to multidisciplinary stroke function improvement service |
| 57306 | other primary cardiomyopathies |
| 59534 | h/o: peripheral vascular disease procedure |

| 63746 | [x]other transnt cerebral ischaemic attacks+related syndroms |
| --- | --- |
| 64837 | dystrophic cardiomyopathy |
| 67640 | puerperal cerebrovascular disorder unspecified |
| 68766 | congestive obstructive cardiomyopathy |
| 70536 | acute cerebrovascular insufficiency nos |
| 73901 | [x]cerebrovascular diseases |
| 89913 | stroke/transient ischaemic attack monitoring telephone invte |
| 91272 | puerperal cerebrovascular disorder with antenatal comp |
| 95347 | [x]other vascular syndroms/brain in cerebrovasculr diseases |
| 96801 | advanced cardiopulmonary resuscitation |
| 97470 | puerperal cerebrovascular disorder - delivered |
| 97780 | arrhythmogenic right ventricular cardiomyopathy |
| 98188 | small vessel cerebrovascular disease |
| 99051 | [x]cardiovascular disease, unspecified |
| 99367 | [x]other cerebrovascular disorders in diseases ce |
| 100015 | transient ischaemic attack clinical management plan |
| 100639 | central post-stroke pain |
| 101251 | [v]personal history of transient ischaemic attack |
| 102326 | suspected transient ischaemic attack |
| 102357 | suspected cerebrovascular accident |
| 104638 | ref multidisciplinary stroke function improvement declined |
| 105520 | admission to stroke unit |
| 105651 | amyloid cardiomyopathy |
| 105738 | carotid territory transient ischaemic attack |
| 106260 | peripheral vascular disease monitoring first letter |
| 106660 | peripheral vascular disease monitoring second letter |
| 109679 | old cerebral infarction on imaging |
| 110337 | [x]sequelae of stroke,not specfd as h'morrhage or infarction |
| 111096 | [x]other specified cerebrovascular diseases |
| 112561 | puerperal cerebrovascular disorder nos |

### Table S4. Severe CKD - Medical codes (CPRD) and ICD-10 codes (HES)

| ICD Code | Description |
| --- | --- |
| Z99.2 | Dependence on renal dialysis |
| Z94.0 | Kidney dialysis |
| Y84.1 | Kidney transplant status |
| T86.1 | Complications of kidney transplant |
| T86.12 | Kidney transplant failure |
| T86.11 | Kidney transplant rejection |
| T86.19 | Other complication of kidney transplant |
| T86.13 | Kidney transplant infection |
| I12.0 | Hypertensive chronic kidney disease with stage 5 chronic kidney disease or end stage renal disease |
| I13.1 | Hypertensive heart and chronic kidney disease without heart failure, with  stage 5 chronic kidney disease, or end stage renal disease |
| I13.2 | Hypertensive heart and chronic kidney disease with heart failure and with stage 5 chronic kidney disease, or end stage renal disease |
| N18.4 | Chronic kidney disease, stage 4 |
| N18.5 | Chronic kidney disease, stage 5 |
| N18.6 | End stage renal disease |
| Z49 | Care involving dialysis |

| Med Code | Description |
| --- | --- |
| 105328 | Cadaveric renal transplant |
| 96133 | Allotransplantation kidney from cadaver, heart non-beating |
| 66705 | Allotransplantation of kidney from live donor |
| 20073 | Renal dialysis |
| 24361 | Transplantation of kidney from cadaver |
| 89924 | Allotransplantation of kidney from cadaver, heart-beating |
| 101756 | Thomas intravascular shunt for dialysis |
| 98364 | Allotransplantation of kidney from cadaver |
| 88597 | Automated peritoneal dialysis |
| 2997 | Transplantation of kidney |
| 64828 | Peritoneal dialysis NEC |
| 71124 | Haemofiltration |
| 11745 | Transplantation of kidney from live donor |
| 30756 | Continuous ambulatory peritoneal dialysis |
| 55151 | Autotransplant of kidney |
| 2996 | Haemodialysis NEC |
| 105787 | Xenograft renal transplant |
| 5911 | [V]Kidney transplanted |
| 109455 | Allotransplantation of kidney from cadaver NEC |
| 70874 | Other specified transplantation of kidney |
| 2994 | Peritoneal dialysis |
| 5504 | Transplantation of kidney NOS |
| 8330 | End-stage renal disease |
| 95508 | Chronic kidney disease stage 5 with proteinuria |
| 11553 | Kidney transplant failure and rejection |
| 6712 | End stage renal failure |
| 53852 | End stage renal failure |

| 26862 | Exploration of renal transplant |
| --- | --- |
| 95405 | Chronic kidney disease stage 5 without proteinuria |
| 18774 | Renal transplant with complication, without blame |
| 17253 | Renal transplant planned |
| 12585 | Chronic kidney disease stage 5 |
| 10081 | Chronic uraemia |
| 512 | Chronic renal failure |
| 54990 | Kidney transplant with complication, without blame |

### Table S5. Ethnicity medcodes used in CPRD

| Medcode | Read term |
| --- | --- |
| White Ethnicity Grouping | |
| 12351 | British or mixed British - ethnic category 2001 census |
| 12352 | English - ethnic category 2001 census |
| 12355 | Greek - ethnic category 2001 census |
| 12402 | Oth White European/European unsp/Mixed European 2001 census |
| 12412 | Italian - ethnic category 2001 census |
| 12421 | Other White background - ethnic category 2001 census |
| 12433 | Baltic Estonian/Latvian/Lithuanian - ethn categ 2001 census |
| 12436 | Scottish - ethnic category 2001 census |
| 12444 | Other white ethnic group |
| 12446 | White British |
| 12467 | Polish - ethnic category 2001 census |
| 12532 | Irish - ethnic category 2001 census |
| 12591 | Other White or White unspecified ethnic category 2001 census |
| 12633 | Other European (NMO) |
| 12681 | Welsh - ethnic category 2001 census |
| 12746 | Turkish - ethnic category 2001 census |
| 12769 | Greek Cypriot - ethnic category 2001 census |
| 22467 | White |
| 24270 | Irish (NMO) |
| 24837 | White Irish |
| 25422 | Albanian - ethnic category 2001 census |
| 26310 | Other white British ethnic group |
| 26341 | Kosovan - ethnic category 2001 census |
| 26391 | Mixed Irish and other White - ethnic category 2001 census |
| 26467 | White Scottish |
| 28866 | Croatian - ethnic category 2001 census |
| 28887 | Cornish - ethnic category 2001 census |
| 28900 | Other mixed White - ethnic category 2001 census |
| 28936 | Other republics former Yugoslavia - ethnic categ 2001 census |
| 28973 | Commonwealth (Russian) Indep States - ethn categ 2001 census |
| 32066 | Turkish/Turkish Cypriot (NMO) |
| 32069 | Turkish Cypriot (NMO) |
| 32126 | Turkish (NMO) |
| 32413 | Turkish Cypriot - ethnic category 2001 census |
| 32778 | Cypriot (part not stated) - ethnic category 2001 census |
| 40102 | Ulster Scots - ethnic category 2001 census |
| 42290 | Gypsy/Romany - ethnic category 2001 census |
| 42294 | Northern Irish - ethnic category 2001 census |
| 45947 | Greek/Greek Cypriot (NMO) |
| 45955 | Greek (NMO) |
| 45964 | Kurdish - ethnic category 2001 census |
| 47074 | Serbian - ethnic category 2001 census |
| 47601 | Irish traveller |
| 47949 | Greek Cypriot (NMO) |
| 55113 | Traveller - ethnic category 2001 census |
| 55223 | Irish Traveller - ethnic category 2001 census |
| 98111 | White British - ethnic category 2001 census |
| 98213 | White Irish - ethnic category 2001 census |
| 99788 | Bulgarian |
| 100143 | Czech |
| Black Ethnicity Grouping | |

| 12350 | African - ethnic category 2001 census |
| --- | --- |
| 12432 | Caribbean - ethnic category 2001 census |
| 12437 | White and Black African - ethnic category 2001 census |
| 12443 | Somali - ethnic category 2001 census |
| 12452 | Black British |
| 12632 | Black Caribbean |
| 12742 | White and Black Caribbean - ethnic category 2001 census |
| 12778 | Black African |
| 24339 | Black, other, non-mixed origin |
| 25451 | Moroccan - ethnic category 2001 census |
| 25623 | Other Black - Black/White orig |
| 25676 | Black - other, mixed |
| 26312 | Black Black - other |
| 32100 | Black Guyana |
| 32136 | Other black ethnic group |
| 32165 | Other Black - Black/Asian orig |
| 32389 | Other Black background - ethnic category 2001 census |
| 32425 | Black Caribbean and White |
| 32443 | Black African and White |
| 32886 | Nigerian - ethnic category 2001 census |
| 35350 | Black - other Asian |
| 35412 | Black - other African country |
| 40096 | Mixed Black - ethnic category 2001 census |
| 40097 | Black British - ethnic category 2001 census |
| 40110 | Black and White - ethnic category 2001 census |
| 41329 | Black N African/Arab/Iranian |
| 46047 | Other Black or Black unspecified ethnic category 2001 census |
| 46812 | Black North African |
| 47028 | North African - ethnic category 2001 census |
| 47950 | Black Caribbean |
| 47965 | Black E Afric Asia/Indo-Caribb |
| 47969 | Other African countries (NMO) |
| 47997 | Black West Indian |
| 48005 | Black Indian sub-continent |
| 49940 | Black and Chinese - ethnic category 2001 census |
| 54593 | Caribbean I./W.I./Guyana (NMO) |
| 57094 | Caribbean Island (NMO) |
| 57435 | Black Caribbean/W.I./Guyana |
| 57752 | Black Arab |
| 57753 | Black East African Asian |
| 57763 | Black Indo-Caribbean |
| 93144 | Guyana (NMO) |
| 99316 | Indo-Caribbean (NMO) |
| South Asian Ethnicity Grouping | |
| 12414 | Indian or British Indian - ethnic category 2001 census |
| 12460 | Pakistani or British Pakistani - ethnic category 2001 census |
| 12482 | Indian |
| 12513 | Other Asian background - ethnic category 2001 census |
| 12608 | Sri Lankan - ethnic category 2001 census |
| 12638 | White and Asian - ethnic category 2001 census |
| 12653 | British Asian - ethnic category 2001 census |
| 12668 | Other Asian ethnic group |
| 12760 | Tamil - ethnic category 2001 census |
| 12887 | Sinhalese - ethnic category 2001 census |
| 24690 | Pakistani |
| 24740 | Bangladeshi |
| 26379 | Other Asian (NMO) |
| 26392 | Punjabi - ethnic category 2001 census |
| 28888 | Bangladeshi or British Bangladeshi - ethn categ 2001 census |
| 28935 | Other Asian or Asian unspecified ethnic category 2001 census |
| 32399 | Caribbean Asian - ethnic category 2001 census |
| 32401 | Other ethnic, Asian/White orig |
| 38097 | E Afric Asian/Indo-Carib (NMO) |
| 39696 | Indian sub-continent (NMO) |
| 46056 | Mixed Asian - ethnic category 2001 census |
| 47077 | East African Asian - ethnic category 2001 census |
| 56127 | Hindu - ethnic category 2001 census |
| 57075 | West Indian (NMO) |
| Other Ethnicity Grouping | |
| 12420 | Filipino - ethnic category 2001 census |
| 12434 | Other - ethnic category 2001 census |
| 12468 | Chinese - ethnic category 2001 census |
| 12473 | Japanese - ethnic category 2001 census |
| 12696 | Other ethnic, mixed origin |
| 12706 | Chinese and White - ethnic category 2001 census |
| 12718 | Chinese |
| 12719 | Vietnamese - ethnic category 2001 census |
| 12730 | Malaysian - ethnic category 2001 census |
| 12756 | South and Central American - ethnic category 2001 census |
| 12757 | Other ethnic group |
| 12795 | Black and Asian - ethnic category 2001 census |
| 12873 | Other Mixed background - ethnic category 2001 census |
| 24272 | Chinese |
| 24962 | N African Arab/Iranian (NMO) |
| 25082 | Iranian (NMO) |
| 25411 | Vietnamese |
| 25937 | Iranian - ethnic category 2001 census |
| 26246 | Latin American - ethnic category 2001 census |
| 26455 | Any other group - ethnic category 2001 census |
| 28909 | Mid East (excl Israeli, Iranian & Arab) - eth cat 2001 cens |
| 30280 | Other ethnic non-mixed (NMO) |
| 32110 | Brit. ethnic minor. spec.(NMO) |
| 32382 | Mauritian/Seychellois/Maldivian/St Helena eth cat 2001census |
| 32408 | Other Mixed or Mixed unspecified ethnic category 2001 census |
| 32420 | Other ethnic, other mixed orig |
| 35459 | Other ethnic, mixed white orig |
| 41214 | Other ethnic NEC (NMO) |
| 46059 | Arab - ethnic category 2001 census |
| 46063 | Jewish - ethnic category 2001 census |
| 46818 | East African Asian (NMO) |
| 46956 | Bosnian - ethnic category 2001 census |
| 46964 | Israeli - ethnic category 2001 census |
| 47005 | Asian and Chinese - ethnic category 2001 census |
| 47091 | Muslim - ethnic category 2001 census |
| 47285 | North African Arab (NMO) |
| 47401 | Other ethnic, Black/White orig |
| 49658 | Sikh - ethnic category 2001 census |
| 50286 | Black Iranian |
| 57764 | Brit. ethnic minor. unsp (NMO) |
| 63872 | Buddhist - ethnic category 2001 census |
| 64133 | Kashmiri - ethnic category 2001 census |
| 89910 | Cook Island Maori |
| Unknown Ethnicity Grouping | |
| 10196 | Ethnic groups (census) |
| 12429 | Ethnic group not given - patient refused |
| 12435 | Ethnic category - 2001 census |
| 12459 | Ethnic category not stated - 2001 census |
| 24340 | Ethnic group not recorded |
| 45199 | Ethnic groups (census) NOS |
| 93749 | Patient ethnicity unknown |

### Table S6. Consultation type codes used to identify consultations in CPRD

| Code | Description |
| --- | --- |
| 1 | Clinic |
| 2 | Night visit, Deputising service |
| 3 | Follow-up/routine visit |
| 4 | Night visit, Local rota |
| 6 | Night visit , practice |
| 7 | Out of hours, Practice |
| 8 | Out of hours, Non Practice |
| 9 | Surgery consultation |
| 11 | Acute visit |
| 18 | Emergency Consultation |
| 27 | Home Visit |
| 28 | Hotel Visit |
| 30 | Nursing Home Visit |
| 31 | Residential Home Visit |
| 32 | Twilight Visit |
| 34 | Walk-in Centre |
| 36 | Co-op Surgery Consultation |
| 37 | Co-op Home Visit |
| 38 | Minor Injury Service |
| 40 | Community Clinic |
| 50 | Night Visit |

### Table S7. Staff role codes used to identify acceptable staff roles for consultations in CPRD

| Code | Description |
| --- | --- |
| 1 | Senior Partner |
| 2 | Partner |
| 3 | Assistant |
| 4 | Associate |
| 5 | Non-commercial local rota of less than 10 GPs |
| 7 | Locum |
| 8 | GP Registrar |
| 9 | Consultant |
| 10 | Sole Practitioner |
| 11 | Practice Nurse |
| 13 | Community Nurse |
| 14 | Midwife |
| 15 | Community Psychiatric Nurse |
| 33 | Other Health Care Professional |
| 34 | Hospital Nurse |
| 36 | School Nurse |
| 38 | Contact Tracing Nurse |
| 45 | Mental Handicap Nurse |
| 54 | Other Nursing & Midwifery |
| 57 | Other Healthcare Scientists |

### Figure S1: Study population flow diagram.

Source population: Total patients in the CPRD GOLD database with

- - age >=18
  - ‘up-to-standard’ registration for ≥ 1 year
  - ‘acceptable’ standard for research
  - at least one T2DM diagnostic code

Between the study start (01/01/2000) and end (31/12/2018) date

**(n=499,393)**

Eligible patients from HES

**(n=150,224)**

T2DM

**n=83,094 (58.8%)**

CHD/Stroke

**n=57,974(41.0%)**

T2DM + CHD/Stroke

**n=262 (0.2%)**

Total eligible patients with HES/ONS linkage data

**(n=141,328)**

Patients with a first diagnosis of T2DM or CHD/Stroke in the study period

**(n=455,311)**

Total patients in the HES database with linkage eligibility **(n=234,944)**

in admitted patient care (for ethnicity)

**(n=218,564)**

Eligible patients with a first diagnosis of T2DM or CHD/Stroke within the study period from CPRD

**(n=197,063)**

Exclusions:

-Cancer at any time before the end of study period

-Previous diagnosis of type 1 diabetes, or gestational diabetes

-Patients diagnosed with severe CKD before/at the index date

**(n=46, 839)**

**(n = 46,839)**

Exclusions:

-Cancer at any time before the end of study period

-Previous diagnosis of cardiovascular disease, type 1 diabetes, or gestational diabetes

-Diagnosis of severe CKD before/at the index date

-Registered at a practice for <1 year at the index date

-Registered at a practice with last collection date before the index date or which is not ‘up-to-standard’ at the index date

**(n = 8,432)**

Please refer to Herret, et al for information regarding CPRD “acceptable” and “Up-to-standard” patients^13^.

### Figure S2. Annual crude consultation rates for primary and secondary care consultation, by exposure status and race/ethnicity


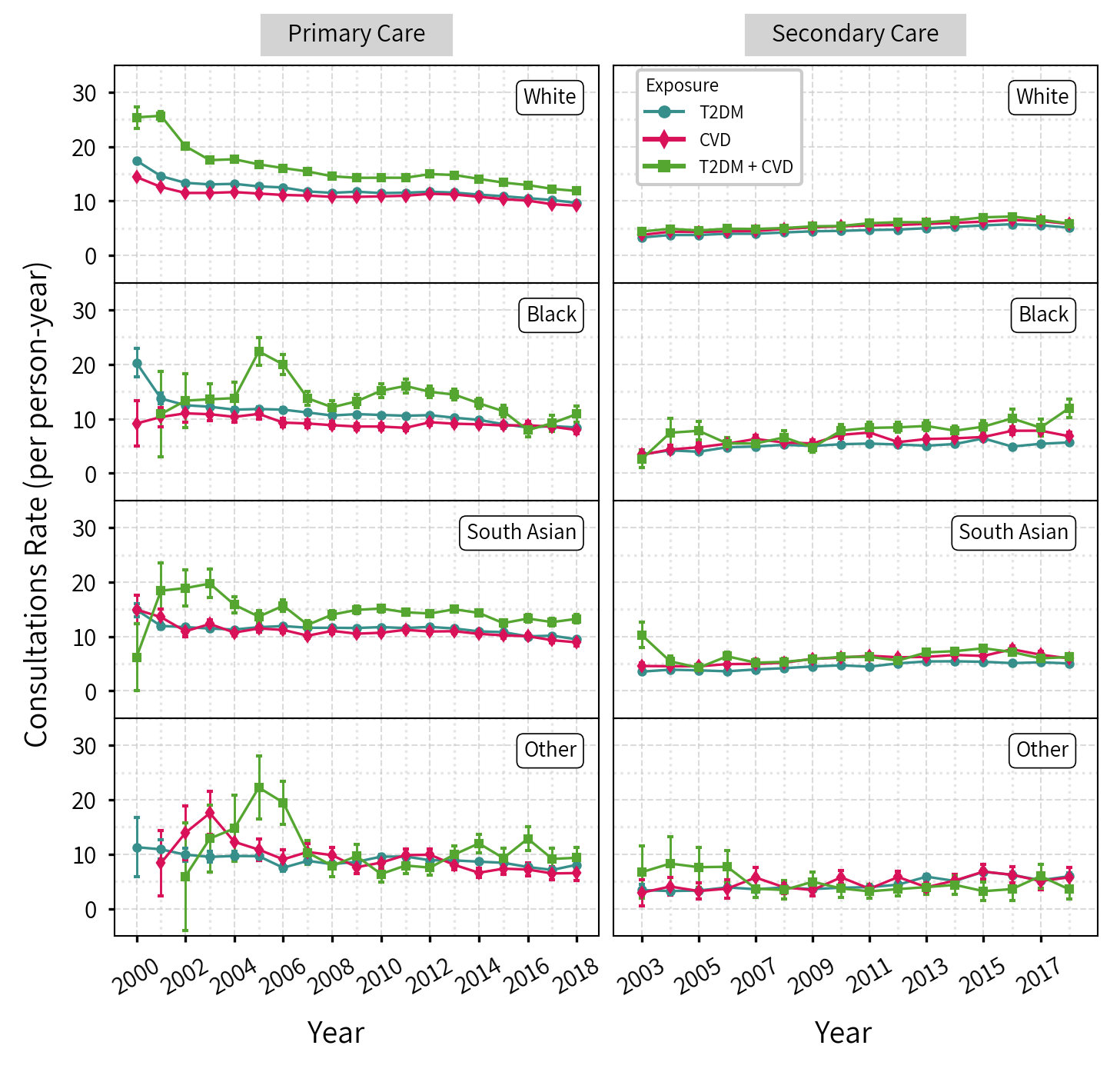


### Figure S3. Annual crude consultation rate for primary and secondary care consultation, by exposure status and sex


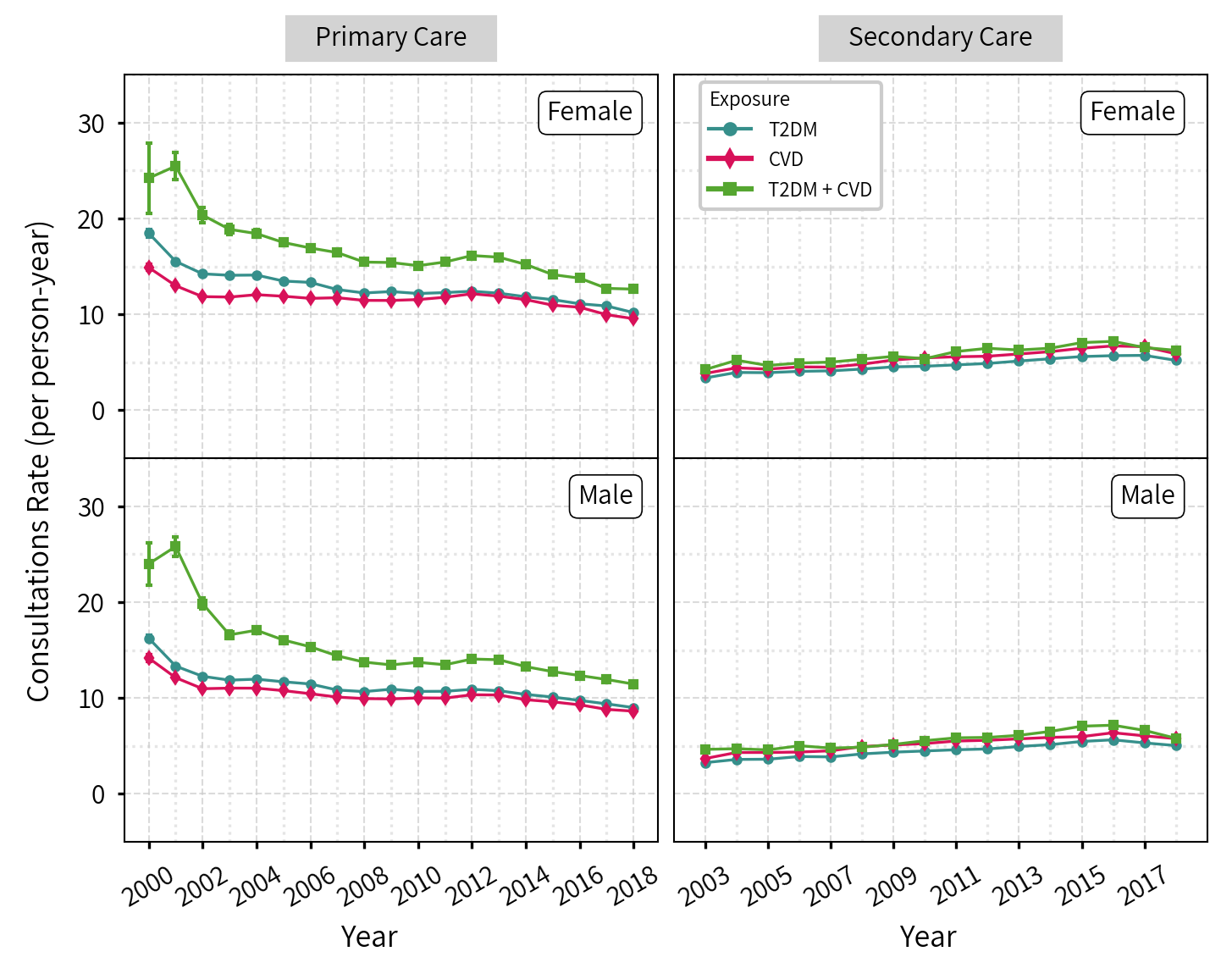


### Figure S4. Annual crude consultation rate for primary and secondary care consultation, by exposure status and age


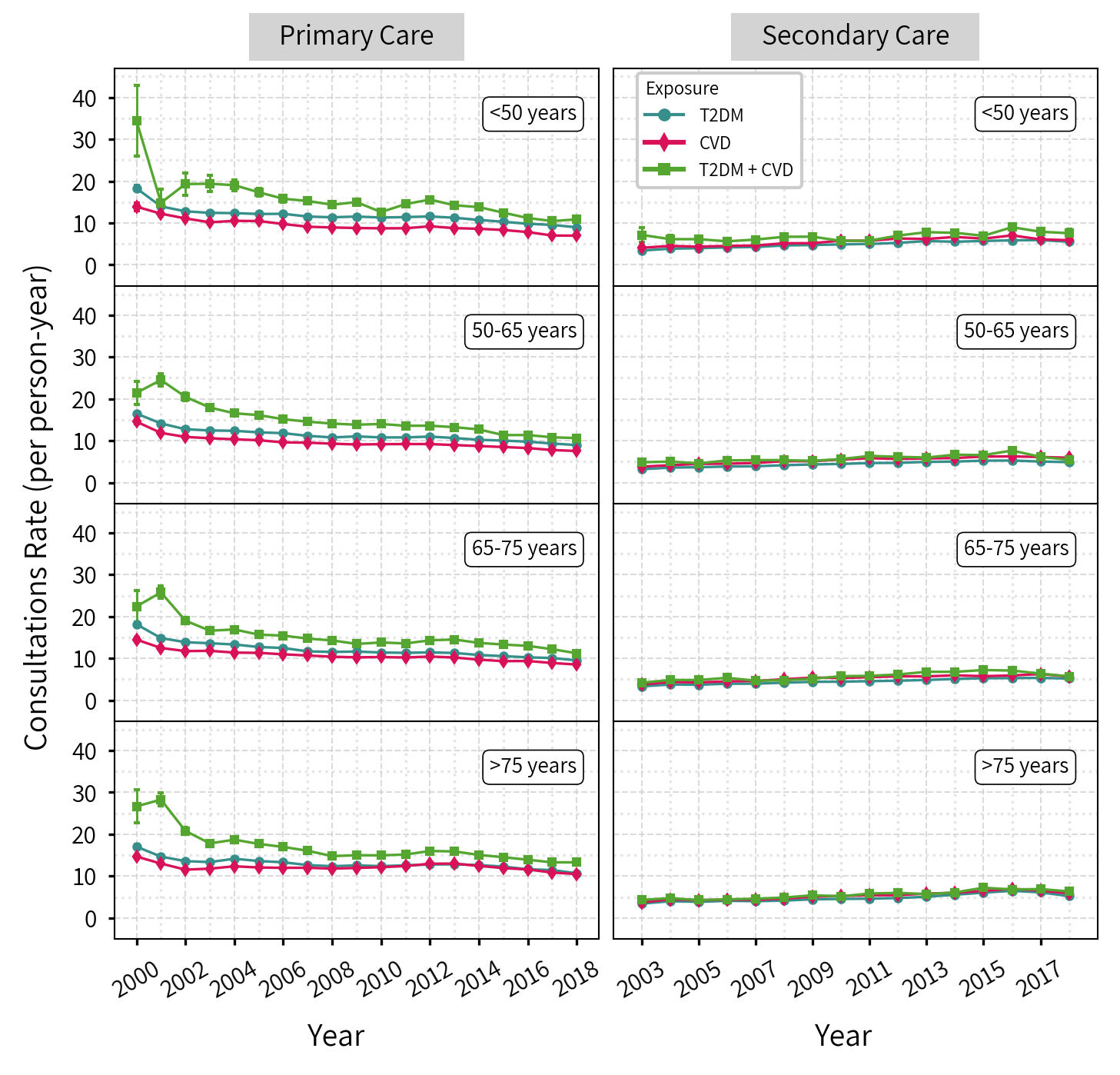


### Figure S5. Mean consultation duration for primary care consultation, by exposure status and sex


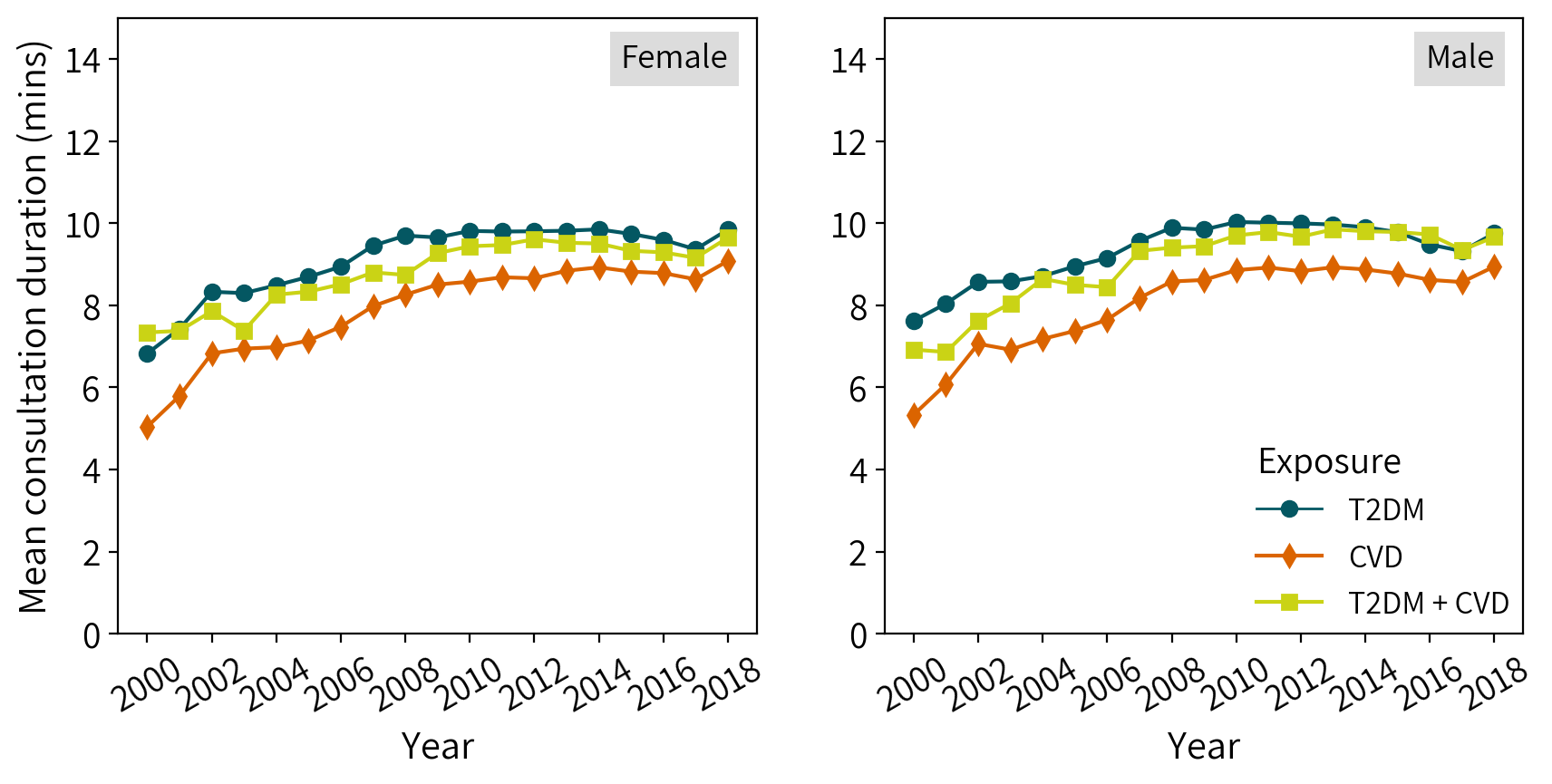


### Figure S6. Mean consultation duration for primary care consultation, by exposure status and ethnicity


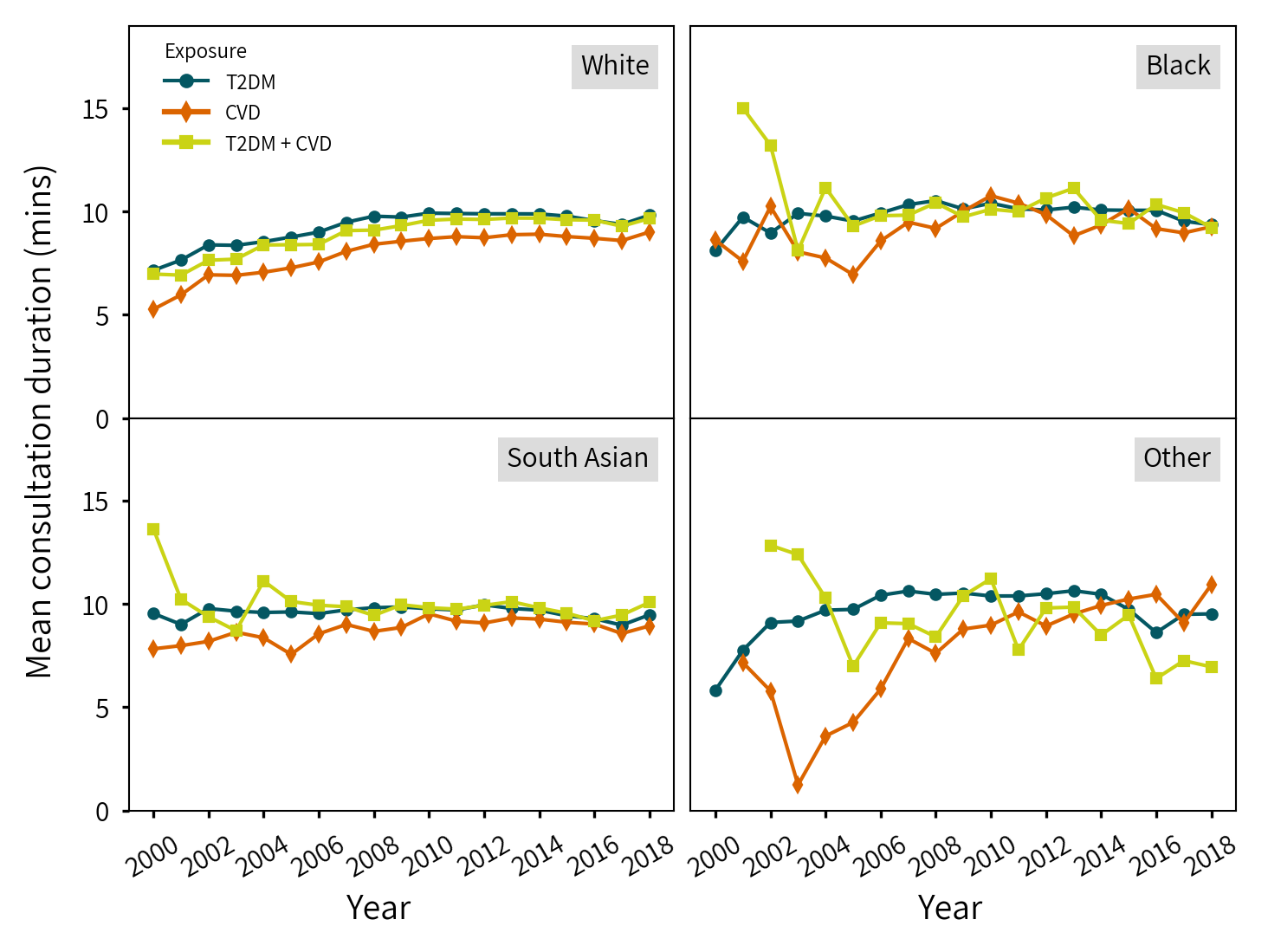


### Figure S7. Mean consultation duration for primary care consultation, by exposure status and age


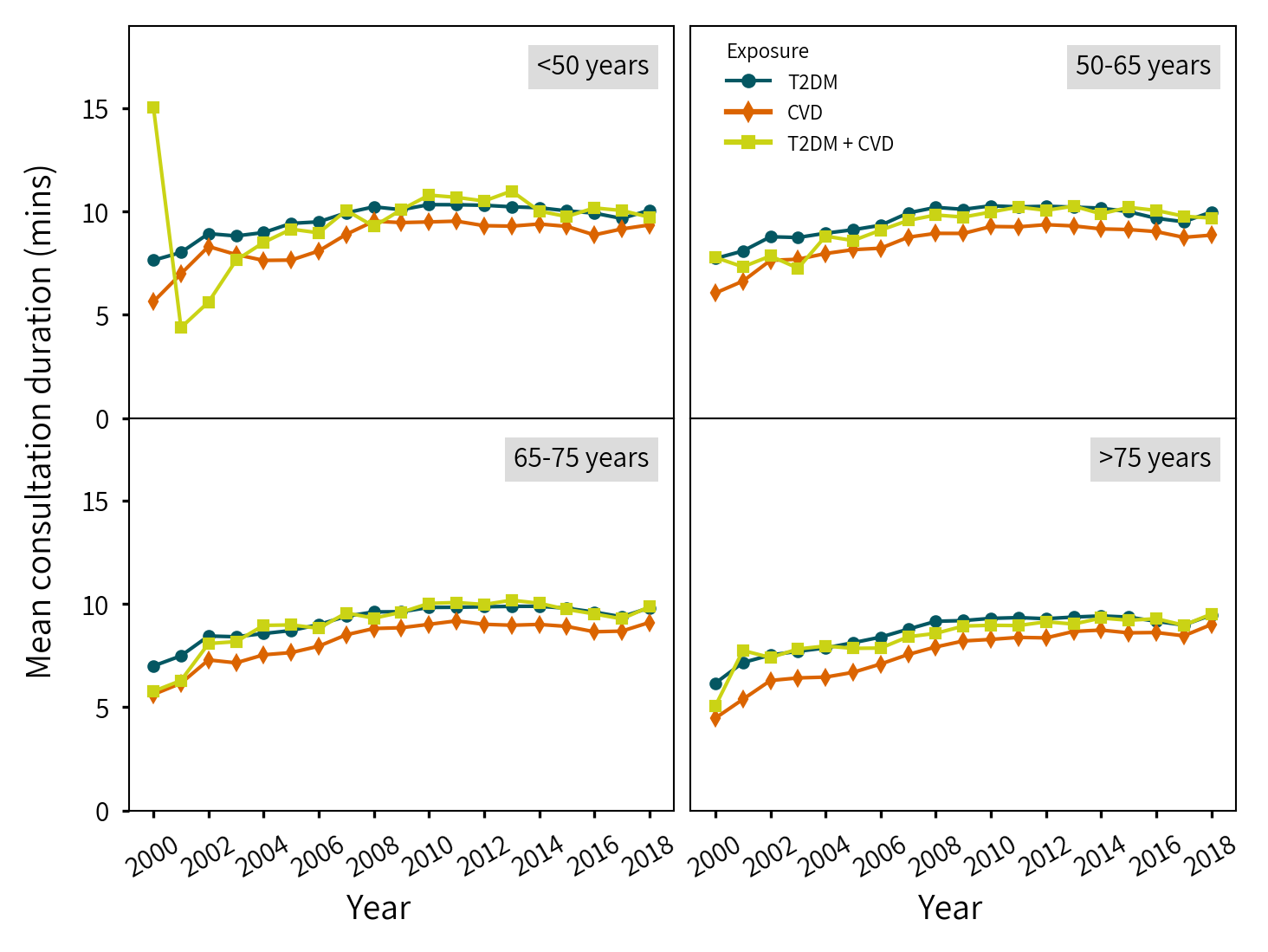

Supplement: Supplementary file 1 — Additional file 1. This file includes additional tables and graphs that further the results of this study. Medcodes and ICD-10 code lists that were used to define variables, outcomes, and exposures in this study have also been included in this file. [file 12933_2021_1435_MOESM1_ESM.docx]
